# Jellyfish mucus-derived organic matter as a source of labile nutrients for the ambient microbial community

**DOI:** 10.7717/peerj.20784

**Published:** 2026-02-12

**Authors:** Nathan Hubot, Sarah L.C. Giering, Neža Orel, Katja Klun, Gerhard J. Herndl, Felix Hohaus, Cathy H. Lucas, Tinkara Tinta

**Affiliations:** 1 The National Oceanography Centre, Southampton, Hampshire, United Kingdom; 2 Marine Biology Station Piran, National Institute of Biology, Piran, Slovenia; 3 Department of Functional and Evolutionary Ecology, Bio-Oceanography Unit, Faculty of Life Sciences, University of Vienna, Vienna, Austria; 4 South Westphalia University of Applied Sciences, Iserlohn, Germany; 5 Ocean and Earth Science, National Oceanography Centre, University of Southampton, Southampton, Hampshire, United Kingdom

**Keywords:** Jellyfish, Mucus, Microbes, Amino acids, Biogeochemical cycling, Coastal ecosystems

## Abstract

Jellyfish are increasingly recognized as a significant contributor to marine organic matter (OM) on a global scale, with implications for ecosystem dynamics. While the role of jellyfish detritus in microbial nutrient cycling has been explored, the contribution of OM released by live jellyfish—primarily as mucus (hereinafter referred to as mucus-associated OM, or MAOM)—remains understudied. This study investigates the release of organic and inorganic nutrients through MAOM from live jellyfish and their effects on ambient microbial communities in the northern Adriatic Sea using a series of leaching and short-term microcosm experiments. Our results show that per gram of MAOM dry weight from the jellyfish *Aurelia* spp, approximatively 2 µmol of phosphate, 4 µmol of dissolved inorganic nitrogen, 18 µmol dissolved organic nitrogen, 134 µmol of dissolved organic carbon and 15 µmol of dissolved free amino acids can be released in the ambient seawater in 24 h. Almost half of the OM is released as dissolved OM (DOM), of which a substantial part is low molecular weight (<1 kDa) molecules. During the first 20 h, the DOM fraction of MAOM was rapidly consumed by the ambient microbial community without a corresponding increase in biomass, likely due to nitrogen limitation. In the subsequent 22 h, microbial growth accelerated to 0.19 ± 0.03 h^−1^ until phosphate became limiting, leading to a sharp decline in microbial production. Our metagenomics analysis revealed that the MAOM-degrading microbial community, dominated by Gammaproteobacteria opportunistic copiotrophs, exhibited increased functional capacity for nutrient assimilation and OM degradation, particularly in the transport and metabolism of amino acids (particularly glycine and taurine) and phosphorus. These traits mirror those found in detritus-degrading microbial communities, suggesting that jellyfish blooms promote the emergence of specialized microbial consortia with shared metabolic capabilities. Taken together, our findings highlight that live jellyfish, through the release of OM, play an active and previously underappreciated role in shaping ambient microbial community dynamics and nutrient fluxes in marine systems affected by jellyfish blooms.

## Introduction

Cnidarians of the subphylum Medusozoa (hereinafter jellyfish) are efficient plankton feeders, incorporating substantial amounts of carbon (C) into their biomass (Luo et al., 2020). Their global biomass is estimated at ∼0.29 Pg C (in the upper 200 m; Luo et al., 2020), accounting for about 16% of the total zooplankton biomass (Wright et al., 2021). Jellyfish blooms can exceed 100 kg of wet weight (WW) m^−3^ over many km^2^ (Lilley et al., 2011), generating diverse ecological and economic impacts (*e.g.*, Richardson et al., 2009). These impacts include interference with fisheries, tourism, and industrial operations, as well as shifts in food web dynamics and biogeochemical cycling—particularly through the production and degradation of jellyfish-derived organic matter (OM; Tinta et al., 2020 and references therein).

At the same time, an increasing number of studies emphasize that jellyfish are integral components of marine ecosystems, fulfilling specific ecological roles beyond their episodic nuisance effects (*e.g.*, Lucas, Loveridge & Hubot, 2024; Brotz et al., 2024). Jellyfish contribute to trophic interactions as both predators and prey, influence nutrient regeneration, and participate in C transport and sequestration (*e.g.*, Hays, Doyle & Houghton, 2018; Luo et al., 2020). Additional work highlights their wider value through ecosystem services and expanding applications in food, pharmaceuticals, cosmetics, and other biotechnological fields (*e.g.*, Graham et al., 2014; Merquiol et al., 2019). This more balanced perspective highlights the importance of understanding the mechanisms through which jellyfish interact with their environment, including the fate of the OM they release.

The biochemical composition and stoichiometry of jellyfish biomass differ substantially from those of phytoplankton and crustacean zooplankton. Phytoplankton OM typically has a C:N ratio of ∼6.6 (Redfield, Ketchum & Richards, 1963) and consists of approximately 40% proteins, 26% carbohydrates, and 15% lipids (Ríos et al., 1998). Crustacean zooplankton show a C:N ratio of 4.8–6.2, with highly variable proportions of proteins (20–70%), lipids (0.5–74%), and other components including chitin and carbohydrates (2–10%; Ventura, 2006). In contrast, jellyfish biomass is composed of 82 ± 4% protein, 11 ± 3% lipids, and 7 ± 4% carbohydrates (Hubot, Giering & Lucas, 2022). Lacking a chitinous exoskeleton and containing less lipids, jellyfish exhibit a high protein-to-lipid ratio (∼3.3) and a low C:N ratio (3.6 ± 0.2; Hubot, Giering & Lucas, 2022), making their OM a protein-rich, high-quality, and easily degradable substrate for marine bacteria (Benner, 2002; Pitt et al., 2013; Tinta, Klun & Herndl, 2021).

It has been shown that different types of jellyfish-derived OM, *i.e.,* excreta and detritus (*i.e.,* carcasses), can alter microbial food web dynamics and play a significant role in C storage and turnover within marine ecosystems (Pitt, Welsh & Condon, 2009; Condon et al., 2011; Guy-Haim et al., 2020). The microbial degradation of jellyfish detritus has been recently studied in detail for two model species: the scyphozoan medusa *Aurelia aurita* s.l. (Tinta et al., 2020; Tinta et al., 2023) and the ctenophore *Mnemiopsis leidyi* (Fadeev et al., 2024). In the northern Adriatic Sea, the decay of an *A. aurita* bloom at ∼10 individuals per m^3^ releases around 100 mg L^−1^ of medusa-derived detrital OM, including ∼44 µmol L^−1^ as dissolved organic carbon (DOC), 13 µmol L^−1^ as total dissolved nitrogen (TDN), 11 µmol L^−1^ of total hydrolyzable dissolved amino acids (THDAA) and 0.6 µmol L^−1^ phosphate (PO_4_^3^−^^; Tinta et al., 2020). In comparison, *M. leidyi* blooms release roughly half these concentrations of DOC, TDN, and PO_4_^3^−^^ into the water column (Fadeev et al., 2024). Both types of detritus supported rapid growth of copiotrophic prokaryotes, but the magnitude and efficiency of the microbial response varied, reflecting differences in OM composition between the two species (Fadeev et al., 2024). Prokaryote communities exposed to medusa detritus exhibited a high growth efficiency (65 ± 27%; Tinta et al., 2020), suggesting efficient incorporation of medusa-derived detrital OM into biomass. In contrast, the microbes degrading ctenophore detritus showed much lower growth efficiency (18–27%; Fadeev et al., 2024), indicating that a larger proportion of ctenophore-derived detrital OM was respired. These contrasting microbial responses highlight the importance of understanding the composition and fate of jellyfish-derived OM, as it has significant implications for the role of jellyfish in oceanic C cycling.

While several studies have examined the composition and fate of jellyfish tissue, far less is known about the mucus they produce. Estimates of mucus release rates are limited to a few species—for example, *Aurelia aurita* s.l. releases ∼1.2 mg C ind^−1^ d^−1^ while *Chrysaora quinquecirrha* produces 0.4–13.4 mg C g DW^−1^ d^−1^ (Hansson & Norrman, 1995; Condon, Steinberg & Bronk, 2010, respectively). Like jellyfish tissue, mucus is protein-rich (80% proteins, 14% lipids, 6% carbohydrates; Hubot, Giering & Lucas, 2022) and dominated by water (∼95% wet weight), with the remainder consisting of mucins (∼3%) and other molecules (∼2%; Bakshani et al., 2018). Chemically, jellyfish mucus consist of organic material such as DOC, dissolved organic nitrogen (DON), and dissolved organic phosphorus (DOP), as well as inorganic nutrients, mainly ammonium (NH_4_^+^) and PO_4_^3^−^^ (Pitt, Welsh & Condon, 2009; Condon et al., 2011). Mucus OM has low C:N ratios, averaging 3.9 ± 0.4 across several scyphozoan species (Hubot, Giering & Lucas, 2022), compared to higher values (8.1 ± 6.2) reported for mucus DOM in *C. quinquecirrha* (Condon et al., 2011). These differences suggest that mucus DOM represents a C-rich pool that may be less bioavailable, together shaping microbial activity and nutrient cycling in the water column. Recent evidence indicates that mucus composition may be broadly conserved across species, but little is known about how it varies with feeding, reproduction, or stress (Savoca et al., 2022). Likewise, its impact on microbial communities remains poorly understood. One study found that jellyfish-derived DOM, including mucus, is rapidly respired by pelagic microbes, with a bacterial growth efficiency of ∼30% (Condon et al., 2011). Better understanding of jellyfish mucus dynamics is essential for accurately incorporating jellyfish into global ocean biogeochemical models.

Here, we characterize and quantify nutrient release from jellyfish mucus (hereafter mucus-associated OM; MAOM) and assess its impact on the structure and functioning of the microbial community in ambient seawater. We hypothesize that (1) MAOM release increases inorganic and organic nutrient concentrations in ambient seawater, and (2) these nutrients are rapidly taken up by the ambient seawater microbial community, leading to shifts in its composition and function. The choice of the jellyfish, *Aurelia* spp., the experimental design and some of the methods were based on a recent study on jellyfish detritus degradation in the same coastal ecosystem (Tinta et al., 2020), allowing for direct comparison and a more comprehensive understanding of the role jellyfish blooms play in coastal ecosystems.

## Materials & Methods

### Sampling

A total of 20 specimens of *Aurelia* spp. were collected along the Slovenian coast of the Gulf of Trieste (northern Adriatic), during the peak and the senescent phase of their spring bloom in 2019 (10 individuals on April 18th) and 2021 (10 individuals on June 16th), respectively. Jellyfish were sampled individually from the surface waters together with ambient seawater using a 10-L plastic bucket, rinsed with ambient seawater prior to sampling. All collected jellyfish were transported to the laboratory while kept in the dark at *in situ* sea surface temperature (14 °C and 21 °C for 2019 and 2021, respectively). In the laboratory, the MAOM was collected as described by Hubot, Giering & Lucas (2022). Briefly, after rinsing the jellyfish by immersion in filtered seawater, specimens were placed in a large acid-cleaned and Milli-Q-rinsed beaker without water. The absence of water induced stress on the jellyfish which triggered mucus excretion. After 10–30 min at room temperature, the jellyfish was removed, and the mucus captured in the beaker was transferred to sterile 50-mL tubes and stored at −20 °C. This method of mucus collection allows for rapid acquisition of dense mucus material. However, stress-induced mucus may differ from naturally produced mucus. Gastric debris, mesoglea, fragments of tentacles and other excreta may be present in the stress-induced mucus. For that reason, we use the terminology MAOM instead of mucus in this article. Still, given the substantial amount of mucus produced and collected for each individual, we are confident that the MAOM we collected was largely composed of mucus.

The majority of the collected MAOM was freeze-dried (at −45 °C for 7 days; hereafter referred to as ‘dry-MAOM’) as previously described (Kogovšek et al., 2014). Dry-MAOM samples were homogenized with a sterilized pestle and agate mortar and pooled to obtain a representative mix of MAOM from the study area. This way we avoided possible biases arising from variations in the size of different individuals within the population and from distinct phases of the bloom. Dry-MAOM was stored at −20 °C in acid and Milli-Q water-rinsed and pre-combusted glass vials until further processed and/or used in experiments as described below. We applied freeze-drying, which is a widespread method to preserve biomolecules (Merivaara et al., 2021) to maintain the biochemical properties of the fresh MAOM. At the same time our approach provided a homogenous sample, ensuring reproducibility of our experiments (Tinta et al., 2020). However, since many studies investigating biological degradation use simple freezing at −20 °C to preserve biomass of interest, we tested the effect of freeze-drying *vs.* freezing on the chemical properties of MAOM. For this purpose, part of collected MAOM was stored at −20 °C until further processed and/or used in our experiments (referred to as ‘frozen-MAOM’, as described below). In addition, the percentage of dry weight (DW) in MAOM WW was estimated by measuring the weight of 8 MAOM samples before and after freeze-drying. Measurements were performed using an ultra-microbalance (Mettler Toledo XPE26, readability: 1 µg).

### Dialysis

A fraction of dry-MAOM was dialyzed using Spectra/Por 7 Membrane tubing (Sulfur and Heavy Metal Free, Spectrum) with a molecular weight cut-off (MWCO) of 1 kDa to determine the ratio between the high- (>1 kDa) and the low- (<1 kDa) molecular weight compounds (HMW and LMW, respectively) of MAOM (as previously described by Tinta et al., 2020). Details of the dialysis procedure are described Supplementary Material. The concentration and composition of the LMW fraction of MAOM was determined by measuring the concentration of DOC and total dissolved nitrogen (TDN) in the dialysate.

### Leaching experiments

The concentration and composition of particulate organic matter (POM) and DOM together with the inorganic nutrients leaching from the dry-MAOM were determined by dissolving 250 mg dry-MAOM powder in 1 L of 0.2-µm filtered aged (∼1 month) seawater (ASW; see next section for details; nutrient composition in Table S1) in a glass Erlenmeyer flask (acid-washed, Milli-Q water-rinsed, and pre-combusted) on a shaker in the dark at *in situ* sea surface temperature (21 °C; as previously described by Tinta et al., 2020). ASW was used to provide a low and consistent chemical background for the incubations. Triplicate experimental flasks were subsampled at 1, 6 and 24 h after the dry-MAOM additions for particulate organic carbon (POC) and particulate organic nitrogen (PON) by collecting and filtering 50 mL onto combusted Whatman GF/F (∼0.7 µm pore size) filters using an acid-washed, Milli-Q water-rinsed, and pre-combusted glass filtration system. The filter was used to determine the concentration of POC, while the filtrate was used to measure DOC, TDN, DFAA and inorganic nutrients. Microbial abundance was determined at the end of the experiment to check for potential contamination.

### MAOM degradation experiment

We studied the response of the ambient seawater microbial community to MAOM in a short-term batch culture experiment. The impact of pre-processing of MAOM was studied by setting up a treatment with frozen- and dry-MAOM in parallel. Both treatments received MAOM enrichments resembling a jellyfish bloom scenario. The mucus release rates by *Aurelia* spp. in the Adriatic Sea were never studied; we hence used data from the Skagerrak strait, *i.e.,* DOC release rate of 1.2 mg C ind^−1^ d^−1^ (Hansson & Norrman, 1995). We assumed that the mucus produced in one day by a dense bloom (50 ind m^−3^) of small jellyfish (9.5 to 18 cm in diameter; Hansson & Norrman, 1995) is equal to 60 µg C L^−1^, which is equivalent to 4 mg DW L^−1^ (based on a mucus C content of 1.5% DW; Hubot, Giering & Lucas, 2022). Therefore, the 5-L incubators of the dry-MAOM treatment received an addition of 20 mg of dry-MAOM each, while the same concentration in the frozen-MAOM treatment was achieved by an addition of 500 mg of frozen-MAOM (equivalent to a concentration of 4 mg of DW L^−1^; based on DW = 4%WW; see Result section). Flasks with no MAOM amendment served as control.

For each treatment (dry-MAOM, frozen-MAOM and control), three acid-washed, Milli-Q water-rinsed and pre-combusted 5-L borosilicate glass flasks were filled up with 0.2-µm filtered ASW and freshly collected 1.2-µm pre-filtered coastal ambient seawater (FSW; serving as microbial inoculum) in a ratio of 9:1 (nutrient composition in Table S1). Seawater for both ASW and the microbial inoculum was collected at 5 m depth in the centre of the Gulf of Trieste using 5-L Niskin bottles connected to a carousel water sampler (SBE 32, Sea-Bird Electronics). Seawater for ASW was collected in May 2021 and aged in acid-washed and Milli-Q water-rinsed 20-L Nalgene carboys for more than a month at room temperature in the dark. Seawater for the microbial inoculum was sampled and filtered on 13 July 2021, the same day as the start of the experiment.

All flasks were incubated in the dark at *in situ* sea surface temperature (21 °C), corresponding to the average warm period near surface sea temperatures (April–September) in the coastal eastern Adriatic region (Vilibić, Dunić & Peharda, 2022). The experimental flasks were gently mixed before each sampling, which took place after 0, 5, 10, 20, 30 and 42 h. By 42 h the microbial community in MAOM treatments reached a late exponential growth phase and the experiment was terminated. At each time point, 50 mL was sampled from the flasks, leaving ∼94% of the initial volume at the end of each experiment. For each sample, we analysed microbial abundance (one replicate was analysed in real-time to follow microbial community growth), prokaryotic heterotrophic production (PHP), DOC, TDN, DFAA and inorganic nutrients (analyses described in detail below). At the end of the experiment, four L of each flask was filtered to collect the microbial biomass for metagenomic analysis (see details below).

#### Microbial abundance

Microbial abundance was determined from 1.5 mL subsamples fixed with formaldehyde (2% final concentration) and stored at −80 °C until processed. For enumerating microbes, one mL of sample was filtered onto a 0.2-µm black polycarbonate filter (Millipore, Burlington, MA, USA; using a glass filtration system and a vacuum pump at low pressure (<200 mbar) and stained with DAPI (4,6-diamino-2-phenylindole, 2 µg mL^−1^, Sigma, Burlington, MA, USA) Porter & Feig, 1980). From each sample, more than 200 cells or at least 10 fields per filter were counted using an Olympus BX51 epifluorescence microscope (1,000x magnification).

#### Prokaryotic heterotrophic production

The PHP was measured in triplicate by ^3^H-leucine incorporation for 1 h (Kirchman, K’nees & Hodson, 1985). ^3^H-leucine was added at 20 nM final concentration (Perkin Elmer, Springfield, MO, USA) to all the samples. Samples containing trichloroacetic acid (TCA, 5% final concentration) served as control. The incubation was stopped by adding TCA (5% final concentration) to all samples, followed by a centrifugation wash (Smith & Azam, 1992). Radioactivity was measured using a liquid scintillation counter (Canberra Packard and TriCarb Liquid Scintillation Analyzer, model 2500 TR). PHP was calculated using the conversion factor for coastal and shelf environments (1.35 kg C mol Leu^−1^; Giering & Evans, 2022).

#### Microbial metagenomes

At the end of the experiment, the microbial biomass was collected by filtering 4 L onto 0.2-µm polyether sulfone membrane filters (47 mm; PALL Inc., New York, NY, USA) from each of the triplicate of the three experimental treatments (frozen-MAOM, dry-MAOM and control) using acid-washed and Milli-Q water-rinsed filtration sets and applying a low (<200 mbar) pressure. Filters were immediately transferred into sterile cryotubes and stored at −80 °C. DNA was extracted from the filters as described by Angel (2012), with slight modifications for extraction from filters (Bayer, Countway & Wahle, 2019) as described by Tinta et al. (2020). The DNA from each of the three treatments was pooled and sent to Microsynth AG (Switzerland) for the construction of shotgun metagenomic DNA libraries (Illumina TruSeq; Illumina, San Diego, CA, USA) and sequencing (Illumina NovaSeq, San Diego, CA, USA; 2 × 150 bp). Raw reads were deposited at the European Nucleotide Archive under the accession number PRJEB66855.

The quality of the paired-end reads was analyzed using FastQC (v0.11.9; Andrews, 2010) and reads were trimmed using Trim-galore (v0.6.6; Krueger, 2020) to remove any low quality read (Phred quality score ≤ 20). The reads were then assembled with the MEGAHIT assembler (v. 1.2.9, Li et al., 2016) using default parameters and allowing a minimum contig length of 800 nt. Assembly statistics were obtained using MetaQuast v. 5.0.2 (Mikheenko et al., 2018). The assemblies were indexed, and the pair-end reads of each sample were mapped back to the contigs of their specific assembly using Bowtie2 (v2.5.1, Langmead & Salzberg, 2012). The outputs were converted to bam files and sorted using Samtools (v1.17, Li et al., 2009). The data were further analysed using anvi’o (v. 7.1, Eren et al., 2021). First, the names of the contigs from the assemblies were simplified using the command anvi-script-reformat-fasta command with the—simplify-names flag, only keeping contigs longer than 1,000 nt. Then, a contig database was created for each sample using the commands anvi-gen-contigs-database. Gene calls in contigs of the assemblies were identified using the Hidden Markov model with the command anvi-run-hmms. Subsequently, genes were annotated with functions from the NCBI’s Clusters of Orthologous Groups of proteins (COGs) database using the command anvi-run-ncbi-cogs. The COG database allows the classification of protein sequences into 17 functional categories (Tatusov et al., 2001). Additionally, the amino acid sequences of the identified genes were extracted from the contigs database using the command anvi-get-sequences-for-gene-calls. The taxonomical composition of the gene calls was annotated using Kaiju v1.7.3 using the refseq database and functional annotation was further investigated using eggnog-mapper (v2.1.10; Cantalapiedra et al., 2021). The gene abundance was calculated as the mean coverage of the contig containing that gene divided by the sample’s overall mean coverage. For taxonomic profiling, the metagenomic 16S rDNA Illumina tags (mitags) were extracted from the metagenomes following the protocol of Logares et al. (2014). The mitags were clustered into OTUs and counted using USEARCH (Edgar, 2010). The OTUs were then mapped at 97% similarities to the taxonomically annotated 16S reference database from the Ribosomal Database Project (RDP; Cole et al., 2014) using USEARCH.

### Chemical analysis

#### Particulate and dissolved organic carbon and nitrogen

For POC and PON analyses, samples of 50 mL were filtered onto combusted 25 mm Whatman GF/F filters using an acid-washed, Milli-Q water-rinsed, and pre-combusted glass filtration system. GF/F filters were stored at −20 °C until analyzed for POC and PON by combustion at 1,150 °C with an elemental analyzer (Vario Micro Cube, Elementar) with a 3% accuracy. For DOC and TDN analyses approximately 30 mL of the GF/F filtrate was collected into acid-washed, Milli-Q water-rinsed, and pre-combusted glass vials and stored at −20 ° C until analysis. DOC and TDN analyses were performed by a high temperature catalytic method using a Shimadzu TOC-L analyzer equipped with a total N unit (Hansell, 1993). The calibration for non-purgeable organic C was done with C_8_H_5_KO_4_ and for TDN KNO_3_ was used. The results were validated with surface sea reference (SSR) water for DOC (CRM Program, Hansell Lab). The precision of the method, expressed as DSR% was <2%. Due to low MAOM additions and small filtered volumes, POC/PON data from the MAOM degradation experiment were inconsistent and disregarded; only leaching experiment data were retained.

#### Dissolved inorganic nutrients

Dissolved inorganic nitrogen (DIN; NH_4_^+^, NO_2_^−^, NO_3_^−^) and PO_4_^3^−^^ concentrations were determined spectrophotometrically by gas-segmented continuous flow analysis (QuAAtro, Seal Analytical) following standard methods (Hydes et al., 2010). The quality control is performed by using certified reference material for nutrients in seawater (KANSO TECHNOS) during analysis and by annually participating in an intercalibration program (QUASIMEME Laboratory Performance Study).

#### Dissolved free amino acid analysis

Samples for DFAA analyses were filtered through combusted Whatman GF/F filters using acid-washed, Milli-Q water-rinsed, and pre-combusted glass filtration systems and analyzed as previously described by Tinta et al. (2020). Briefly, approximately four mL of the GF/F filtrate was collected in dark glass vial and stored at −20 °C until analysis. A volume of 500 µL of sample was directly pipetted into acid-washed, Milli-Q water-rinsed, and pre-combusted glass ampules, and analyzed by a Shimadzu Nexera X2 ultra high-performance liquid chromatograph with a fluorescence detector (RF-20A XS). Pre-column derivatization was applied with ortho-phthalaldehyde according to the protocol of Jones, Pääbo & Stein (1981).

### Statistical analysis

The differences in the cumulative amount of nutrients leaching from frozen- and dry-MAOM after 24 h were investigated using Wilcoxon rank-sum non-parametric tests. Furthermore, the statistical differences between the measured parameters in triplicate from the MAOM degradation experiment were assessed using Wilcoxon rank-sum non-parametric tests at each time point. First, differences between the two MAOM treatments were analysed at each time point. Then, the combined data from both MAOM treatments were compared with the control treatment at each time point. The DFAA composition throughout the MAOM degradation experiment was analyzed using multivariate ordination from the vegan package in R (Oksanen et al., 2013; R Core Team, 2022). Further, Bray–Curtis dissimilarities were calculated and visualized using non-metric multidimensional scaling (NMDS) followed by the metaMDS function with a stress level cut-off value <0.2 (Legendre & Legendre, 1998). Differences in DFAA composition and microbial community composition at the end of MAOM degradation for the treatments (dry-MAOM, frozen-MAOM and control) were tested using permutational multivariate analysis of variance (PERMANOVA; a non-parametric alternative for the multivariate analysis of variance; Anderson, 2017) using the adonis2 function. Differences in group homogeneities were further tested using the function betadisper (Anderson, Ellingsen & McArdle, 2006) and ANOVA. Results were considered significantly different at *p* < 0.05 and all statistical analyses were done using R version 4.2.2 (R Core Team, 2022).

## Results

### MAOM as source of organic and inorganic nutrients

Two series of leaching experiments showed that during the first hour following the addition of MAOM to the ASW, the OM in both frozen- and dry-MAOM on average consisted of 56 ± 14% of POC and 44 ± 14% of DOC (Table S2, Fig. S1). Over the 24 h incubation, an average of 258.8 ± 204.5 µmol of DOC, 44.7 ± 34.7 µmol of TDN, 4.3 ± 2.1 µmol of DIN, 40.3 ± 33.2 µmol of DON (TDN-DIN), 25.5 ± 18.3 µmol of DFAA and 1.9 ± 0.7 µmol of PO_4_^3^−^^ were released per g of DW from frozen- and dry-MAOM (Table 1). Glycine was the most abundant DFAA released, accounting for 53.6 ± 8.3 mol% of the total MAOM DFAA pool, followed by taurine, at 8.0 ±  1.0 mol% (Table S3). The difference between the concentrations of DOC, TDN, and DON in the dialysate of dry-MAOM (<1 kDa MWCO membrane; Table S4) and the total dry-MAOM pool indicates that approximately half of the DOC (48 ± 35%) consists of LMW compounds, while nearly all (∼100%) of the TDN and DON is present in LMW form (Table S5). We found that the dissolved fraction of MAOM is relatively C-rich (C:N = 6.7 ± 1.0), while the particulate fraction of MAOM is relatively C-poor (C:N = 3.5 ± 1.0).

After freeze-drying, the dry weight of MAOM averaged only 4% of its original wet weight (Table S6). When normalized to dry weight, the release of dissolved compounds from frozen-MAOM was up to 3.5 times higher than from dry-MAOM, with variability (*i.e.* standard deviation) up to nine times greater (Table 1). Despite this, no statistically significant differences in the release of dissolved nutrients were observed between the two treatments, except for total dissolved nitrogen (TDN), which was significantly higher in the frozen-MAOM (Wilcoxon test, *p* < 0.05; Table 1). These results indicate that the choice between freezing and freeze-drying does not substantially alter the nutrient-releasing potential of MAOM, suggesting that its key biochemical properties are preserved across pre-processing methods—an important consideration for marine research and applied studies. However, it is important to consider that both freezing and freeze-drying can alter the humoral immune properties of jellyfish mucus (Cordero et al., 2016), which may have modified the immunological characteristics of the MAOM in our study and, consequently, influenced microbial community dynamics during its degradation. Furthermore, the higher variability observed in nutrient release from frozen-MAOM underscores the benefits of freeze-drying followed by homogenization, which offers greater consistency between samples. In addition to improved reproducibility, using dry material also provides practical advantages in terms of handling, storage, and transport.

**Table 1:** Cumulative nutrient release from dry-MAOM. Cumulative amount of dissolved nutrients (NH${}_{4}^{+}$, NO${}_{3}^{-}$, NO${}_{2}^{-}$, PO${}_{4}^{+}$, DIN, TDN, DON, DOC, DFAA) that leached from dry-MAOM within 24 h and *p* values from the Wilcoxon rank-sum tests evaluating the difference between the amount of nutrient released in each leaching experiment.

	Leaching dry-MAOM (µmol gDW^−1^ d^−1^)	Leaching frozen-MAOM (µmol gDW^−1^ d^−1^)	Wilcoxon test *p*
NH_4_^+^	0–3.2	2.4–6.5	0.67
NO_3_^−^	2.2–2.7	0.1–0.4	0.33
NO_2_^−^	0.1–0.1	0.1–0.1	0.33
PO_4_^3^−^^	1.7–1.7	1.5–3.0	1.00
DIN	2.2–5.5	2.9–6.8	0.67
TDN	17.3–26.2	40.4–94.8	0.03^*^
DON	15.1–20.2	37.5–88.1	0.33
DOC	106.3–161.7	208.0–559.2	0.33
DFAA	13.3–16.9	19.0–52.8	0.33

**Notes.**

Values marked with an asterisk (*) indicate statistically significant results (*p* < 0.05).

### Microbial processing of the DOM released by live jellyfish

At the start of the MAOM degradation experiment, the average microbial abundance across all treatments (*n* = 9) was 50.1 ±  7.7 × 10^3^ cells mL^−1^. As no significant differences in microbial abundance, PHP, DOC and TDN were observed between the dry- and frozen-MAOM treatments at any time point (Table S8), the results are presented here as a single MAOM treatment for clarity.

**Figure 1:**
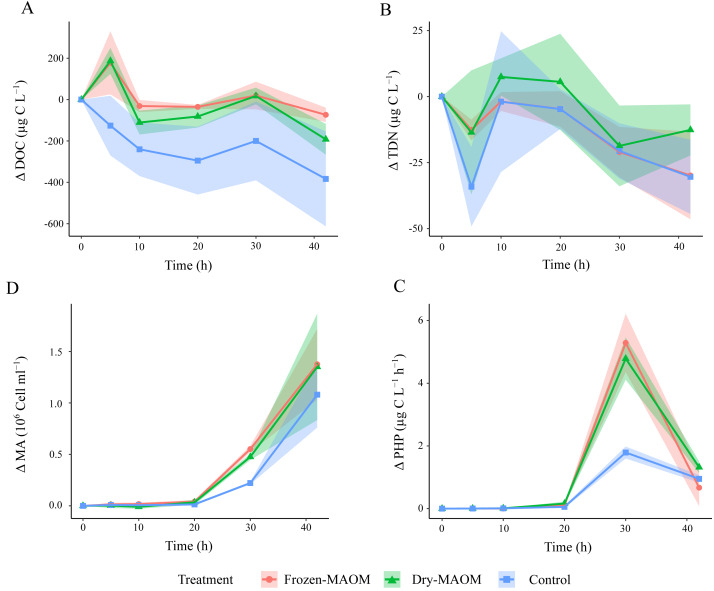
Changes in microbial parameters over time during the degradation experiment. Changes in DOC (A), the TDN (B), the MA (C) and the PHP (D) in the frozen-, dry-MAOM and control treatments. Values are normalized to the value at time = 0. Lines represent the means (*n* = 3) with the coloured area showing the standard deviation.

**Figure 2:**
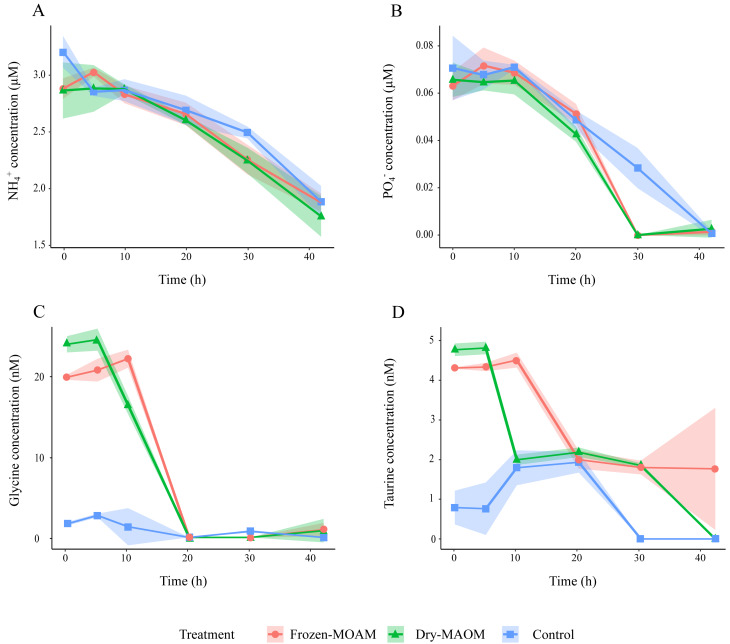
Changes in organic and inorganic nutrient concentrations over time during the degradation experiment. Concentration of NH${}_{4}^{+}$ (A), PO${}_{3}^{-}$ (B), glycine (C) and taurine (D) in the frozen-, dry-MAOM and control treatments. Lines represent the means (*n* = 3) with the coloured area showing the standard deviation.

During the first 5 h, DOC concentrations in the MAOM treatments (*n* = 6) increased significantly compared to the controls (*n* = 3), which showed a decline (Table 2; Fig. 1A). TDN concentrations decreased by 20.1 ± 17.6 µg L^−1^ during the first 5 h in all treatments, followed by an increase of 21.3 ±  14.8 µg L^−1^ in the subsequent 5 h (Fig. 1B). Exponential microbial growth began after 20 h of incubation, with both microbial abundance and PHP peaking at 30 h (Figs. 1C and 1D). At this point, values in the MAOM treatments were significantly higher than in the controls (Table 2), with microbial growth rates of 0.19 ± 0.03 h^−1^ and 0.15 ± 0.02 h^−1^, respectively. Between 20 and 30 h, DOC increased by 83.1 ± 61.7 µg C L^−1^ in all treatments (Fig. 1A). NH_4_^+^ and PO_4_^3^−^^ concentrations gradually declined in both treatments (Fig. 2), while nitrate (NO_3^−^_) and nitrite (NO_2^−^_) remained largely unchanged (Fig. S2). At 30 h, NH_4_^+^ and PO_4_^3^−^^ concentrations were significantly lower in the MAOM treatments compared to the controls (Table 2; Fig. 1B) and PO_4_^3^−^^ was totally depleted at 40 h (Fig. 2B). The incubation was terminated after 42 h, when PHP declined to 0.9 ± 0.4 µg C L^−1^ h^−1^ and microbial abundance peaked at 1.3 ± 0.4 × 10^6^ cells mL^−1^ across all treatments.

**Table 2:** DOC, nutrients, and microbial activity: MAOM *vs.* control. Comparison of DOC, microbial abundance (MA), PHP, NH${}_{4}^{+}$ and PO${}_{4}^{{3}^{-}}$ between MAOM and control treatments at different time points during the MAOM degradation experiment. Only results with significant differences, as determined by Wilcoxon rank-sum tests (*p* < 0.05; Table S6), are presented.

Time (h)	Parameters	MAOM	Control	Wilcoxon test *p*
5	DOC (µg C L^−1^)	182.3 ± 104.5	125.9 ± 144.4	0.02
30	MA (10^4^cell mL^−1^)	56.5 ± 4.2	26.9 ± 1.1	0.02
30	PHP (µg C L^−1^ h^−1^)	4.4 ± 0.7	1.6 ± 0.7	0.01
30	NH_4_^+^(µmol gDW^−1^)	2.2 ± 0.1	2.5 ± 0.1	0.02
30	PO_4_^3−^ (µmol gDW^−1^)	0.0 ± 0.0	0.1 ± 0.1	0.02

At the start of the MAOM degradation experiment, the overall DFAA composition of both MAOM treatments differed significantly from the control (PERMANOVA: *F*_(1,8)_ = 118.9, *R*^2^ = 0.94, *p* < 0.01; Fig. S3), mainly due to high concentrations of glycine, taurine, tyrosine, arginine, alanine, lysine and leucine in the MAOM treatments (Fig. S3). Glycine concentrations showed the fastest decrease (1.5 ± 0.2 nmol L^−1^ h^−1^ between 5 and 20 h), reaching 0 nM after 20 h in all MAOM treatments (Fig. 2C). Taurine concentrations in the MAOM treatments decreased from 4.6 ±  0.3 nM at 5 h to 0.9 ± 1.4 nM at 42 h (Fig. 2D). At the end of the experiment, the DFAA composition differed significantly among the treatments (PERMANOVA: *F*_(1,8)_ = 118.9, *R*^2^ = 0.94, *p* < 0.01; Fig. S3).

### Taxonomic profiling of the microbial community exposed to MAOM

**Figure 3:**
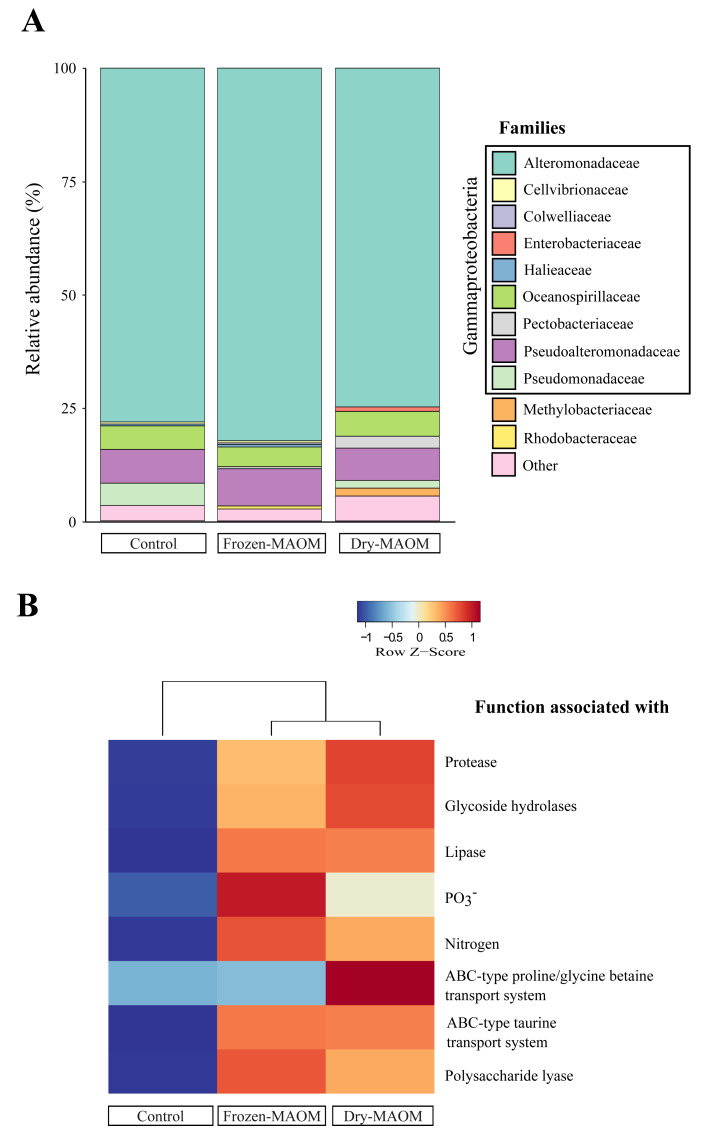
Microbial community and functional profiles after MAOM degradation. Relative abundance of the main microbial families (A) and heatmap of COG functional genes associated with some nutrient assimilation processes (B) from pooled microbial metagenomes at the end of the MAOM degradation experiment.

Taxonomic profiling of microbial metagenomes collected at the end of the MAOM degradation experiment revealed that all communities (*n* = 3) were dominated by Gammaproteobacteria (95 ± 2%), with Alteromonadaceae as the most abundant family (78 ± 4%; Fig. 3A). Pseudoalteromonadaceae were consistently the second most abundant (7 ± 1%), while Vibrionaceae were present at low levels (0.05 ± 0.04%). In addition, Pseudomonadaceae were relatively abundant (5% and 2% for the dry- and frozen-MAOM treatment, respectively) compared to the control (0.1%). The overall relative abundances of bacterial families did not differ significantly between the MAOM treatments and the control (PERMANOVA, *p* = 0.33).

### Functional profiling of microbial community exposed to OM released by live jellyfish

The functional profiling of the microbial communities’ metagenomes at the end of the MAOM degradation experiment indicated that the relative gene abundances in all 17 functional categories of the COGs database were higher in the MAOM treatments relative to the control, except for the categories ‘Extracellular structures’, ‘Cell motility’ and ‘Chromatin structure and dynamics’ (Fig. S4). Genes classified into the ‘Amino acid transport and metabolism’ category were 27 ± 2% more abundant in the MAOM treatments compared to the control. The relative abundance of genes for ABC-type transporters of glycine and taurine was 22 ± 20% and 49 ± 0% higher in the MAOM treatments than in the control (Fig. 3B). Additionally, genes related to P and N metabolism (*e.g.*, the *GlnK* gene coding for the PII N regulatory protein and the *PhoU* gene coding for the PO_4_^3^−^^ uptake regulator protein) exhibited a 42 ± 15% and 42 ± 4% higher relative abundance in the MAOM than in the control (Fig. 3B). Protein degradation-related genes (*e.g.*, the *HflC* protease modulator gene and the ATP-dependent proteases genes *ClpA* and *ClpYQ*) had a 30 ± 5% higher relative abundance in MAOM treatments compared to the control. Based on the CAZy database, glycoside hydrolase and polysaccharide lyase genes had a 24 ± 3% and 87 ± 12%, respectively, higher relative abundance in the MAOM treatments as compared to the control (Fig. 3B).

## Discussion

Jellyfish are increasingly recognized as an important source of OM for the marine ecosystem (Tinta, Klun & Herndl, 2021), yet the chemical composition and the impact of the mucus they produce and release during their life span on the structure and function of the ambient marine food web is not well understood. We characterized the chemical composition of the MAOM of a cosmopolitan coastal bloom-forming jellyfish, the scyphozoan *Aurelia* spp., and used it as a proxy to investigate how jellyfish mucus affects the structure and functioning of the ambient microbial community.

### MAOM as a significant source of DOM for marine microbes

Our results show that MAOM is a substantial source of labile OM, rapidly releasing a diverse suite of dissolved compounds into seawater. A significant portion of this material consists of LMW compounds, which can be readily assimilated by bacteria (Berggren et al., 2010) suggesting that the dissolved fraction of MAOM might provide a readily available material to the heterotrophic microbial community. In addition, the stoichiometric contrast between the C-rich dissolved fraction (C:N = 6.7 ± 1.0) and the nitrogen-rich particulate fraction (C:N = 3.5 ± 1.0), suggests that MAOM DOM may favor microbial respiration over biomass production due to nitrogen limitation.

The high abundance of glycine in the MAOM DFAA pool (53.6 ±8.3%) is likely related to its role as a major osmolyte in marine invertebrates, where it contributes to cellular osmoregulation and volume regulation (Podbielski et al., 2022). In addition, glycine may also derive from the turnover of structural proteins such as collagen or glycoproteins present in jellyfish mucus (Feller et al., 1990). Glycine uptake began between 5 and 10 h after MAOM addition and was rapidly and completely depleted by 20 h, indicating strong microbial demand. Glycine is a key metabolite for many marine heterotrophic bacteria, readily used as both a C and nitrogen source (Kikuchi et al., 2008; Boysen et al., 2022). Its rapid consumption suggests that the microbial community was well-adapted to exploit glycine-rich inputs, and its depletion may have contributed to the observed decline in PHP at 30 h.

The second most dominant DFAA in MAOM was sulfonic acid taurine (8.0 ± 1.0% of the DFAA pool), which, like glycine, plays an important role as an osmolyte, contributing to tissue osmolarity (Welborn & Manahan, 1995). In mucus, taurine may serve a similar osmotic function by helping to retain water and maintain balance, thereby preserving hydration and fluidity in seawater. Once released, taurine is also of microbial interest: many marine bacteria possess specialized transporters and catabolic pathways that allow them to use taurine as a source of sulphur and nitrogen under limiting conditions (Clifford et al., 2019).

Together, the high concentrations of glycine and taurine in MAOM likely reflect their physiological roles as major osmolytes in marine invertebrates, with mucus release providing a pathway for their export to the surrounding water. In dissolved form, these molecules constitute labile metabolites that can stimulate microbial uptake and potentially influence community structure and activity in the water column.

### MAOM is mostly respired by the ambient microbial community

Our results indicate that MAOM is primarily respired rather than converted into microbial biomass. During the first 10 h of incubation, DOC was rapidly consumed without a corresponding increase in PHP, suggesting that the available C supported respiration rather than growth (Figs. 1B, 1C). This pattern is consistent with previous observations showing that jellyfish-derived DOM stimulates microbial respiration within just 6 h (Condon et al., 2011). In deoxygenated ecosystems with large jellyfish biomass, such as the northern Benguela upwelling system (Lynam et al., 2006), this mechanism may exacerbate oxygen depletion, in a manner similar to the impacts reported for jelly-falls on the seafloor (Guy-Haim et al., 2020).

The low PHP during the first 20 h of our experiment may also reflect microbial adaptation to the diluted seawater matrix (9:1 aged seawater to natural seawater), highlighting the need for an adaptation period in future experiments. However, prior work using the same design with jellyfish and ctenophore detritus did not observe such a delay in microbial activity (Tinta et al., 2020; Fadeev et al., 2024), suggesting that the substrate type—mucus *versus* tissue—plays a more critical role than dilution in shaping microbial responses.

Despite its lower nutritional quality, MAOM represents a relevant source of PO_4_^3^−^^, which was rapidly consumed by the microbial community. The drop in PHP following the consumption of PO_4_^3^−^^ suggests that microbial productivity became limited by PO_4_^3^−^^ availability, consistent with known high P requirements and uptake efficiency in heterotrophic prokaryotes (Vadstein et al., 2003; Godwin & Cotner, 2015). In P-limited regions such as the northern Adriatic Sea (Marini & Grilli, 2023), where jellyfish blooms are recurrent (Kogovšek et al., 2018), jellyfish-derived PO_4_^3^−^^ may play a key role in sustaining microbial activity and influencing C cycling.

### MAOM degradation is facilitated by a consortium of opportunistic bacteria exhibiting specific functional traits

The lack of statistical difference between the family composition of microbial communities in the control and the MAOM treatments at the end of the MAOM degradation experiment suggests that the final community composition was driven more by the availability of labile DOM in the enclosed environment rather than by the presence of MAOM. Further, the dominance of Gammaproteobacteria in all treatments (95 ± 2% of the community; Fig. 3) is typical of artificial confinements and enrichment incubation experiments, where opportunistic copiotroph bacteria are favoured (*e.g.*, Eilers, Pernthaler & Amann, 2000; Dinasquet et al., 2013) and contrasts with the ambient Alphaproteobacteria-dominated community typically found in the Gulf of Trieste (Tinta et al., 2015; Orel et al., 2021; Celussi et al., 2024) and of coastal assemblages (*e.g.*, Eastern Mediterranean Sea—(Haber et al., 2022); Ria de Vigo NW Spain—Costas-Selas et al., 2023). Marine copiotrophs such as Alteromonadaceae and Pseudoalteromonadaceae (78 ± 4% and 7 ± 1%, respectively; Fig. 3A) can rapidly metabolize DOC and nitrogen-rich compounds present in seawater therefore inducing a bacterial community shift (Dinasquet et al., 2013). In addition, the enrichment of Pseudomonadaceae in the MAOM treatments (5% and 2% for the dry- and frozen-MAOM treatment, respectively) compared to the control (0.1%), suggests that MAOM provides nutrient- and particle-rich microhabitats favouring opportunistic copiotrophs whose extracellular enzymes might enable rapid colonization and degradation of protein- and polysaccharide-rich MAOM, consistent with the metabolic versatility of marine Pseudomonadaceae (Girard et al., 2023).

The increase in DOC between 20 and 30 h of incubation (8.3 ± 6.2 µg C L^−1^ h^−1^) likely reflects microbial enzymatic degradation of POM, releasing DOM from MAOM. Bacteria typically rely on extracellular enzymes to break down HMW compounds into smaller, utilizable forms (Arnosti, 2011). This is supported by the metagenomic data, which showed elevated abundances of genes involved in protein, lipid, and carbohydrate catabolism in the MAOM treatments, including proteases (30 ± 5%), lipases (36 ± 1%), glycoside hydrolases (24 ± 3%), and polysaccharide lyases (87 ± 12%; Fig. 3B). These enzymatic profiles align with the macromolecular composition of jellyfish mucus—rich in proteins (80%), lipids (14%), and carbohydrates (6%; Hubot, Giering & Lucas, 2022)—indicating active microbial processing of mucus substrates. In particular, the high abundance of glycoside hydrolase genes suggests microbial adaptation for breaking down complex glycan linkages within the mucins (Glover, Ticer & Engevik, 2022) constituting jellyfish mucus.

Overall, the metagenomic analysis revealed a broad shift in microbial functional potential in response to MAOM addition. Compared to the control, MAOM treatments showed higher relative abundances of genes across nearly all COG categories (except Extracellular structures, Cell motility, and Chromatin structure and dynamics; Fig. S4). Notably, genes related to amino acid and inorganic ion transport and metabolism were elevated (27 ± 2% and 22 ± 1%, respectively), reflecting enhanced microbial capacity to assimilate jellyfish-derived nutrients such as DFAA, NH_4_^+^, and PO_4_^3^−^^. For example, ABC-type transporter genes for glycine and taurine were more abundant in MAOM treatments (increased by 22 ± 20% and 49 ± 0%, respectively), consistent with the dominance of these amino acids in the MAOM DFAA pool (53.6 ± 8.3% and 8.0 ± 1.0%) and their rapid microbial uptake (Figs. 2C & 2D). Likewise, genes associated with PO_4_^3^−^^ metabolism increased by 42 ± 15%, correlating with the observed PO_4_^3^−^^ drawdown in these treatments (Fig. 2B). These functional shifts mirror those reported during jellyfish and ctenophore carcass degradation (Tinta et al., 2023; Fadeev et al., 2024), suggesting that MAOM elicits microbial metabolic responses comparable to those triggered by jellyfish detritus.

### Complementary roles of MAOM and jellyfish detritus in fuelling marine ecosystems

**Figure 4:**
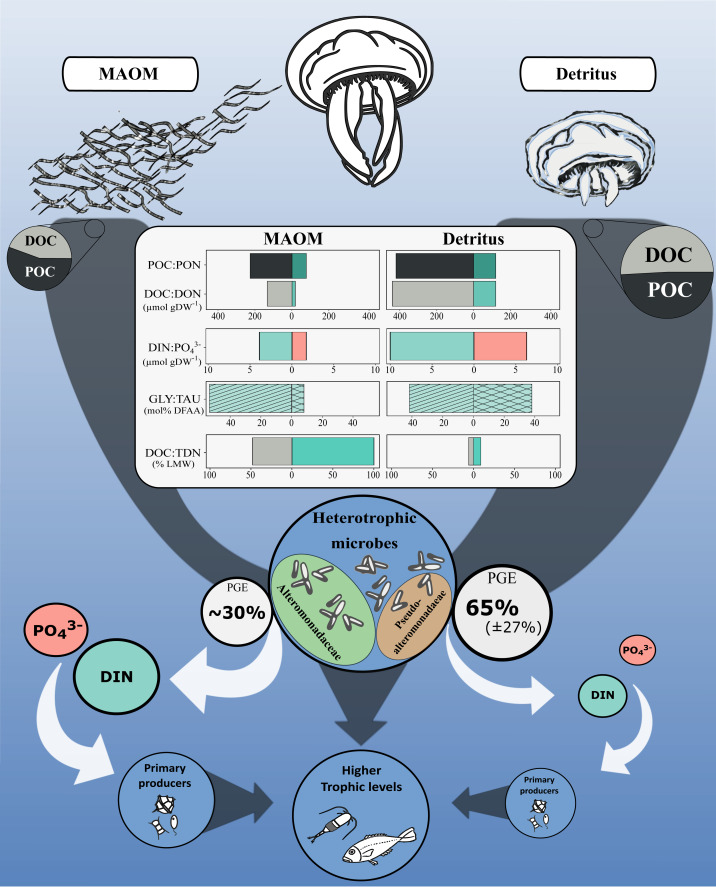
Conceptual diagram of the impact of the microbial degradation of MAOM and detritus on coastal marine ecosystems. Central table compares nutrients (organic and inorganic) released per g of DW. Grey and white arrows represent organic and inorganic flow of nutrients, respectively. Turquoise and light red represent nitrogenous and phosphorous compounds, respectively. GLY, glycine; TAU, taurine; PGE, prokaryotic growth efficiency. Jellyfish detritus data is from Tinta et al. (2020). Mucus PGE is from Condon, Steinberg & Bronk (2010).

Jellyfish blooms introduce substantial OM into marine systems *via* two main pathways: mucus release and post-mortem body degradation (detritus; Fig. 4). While both MAOM and detritus-derived OM contribute similar proportions of POC (56 ± 14% and 49 ± 8% of total OM, respectively), their dissolved fractions differ markedly in composition and bioavailability. MAOM DOM is significantly enriched in LMW compounds (<1 kDa), comprising ∼48% of DOC and nearly all TDN, compared to only 6% and 9%, respectively, in detritus DOM (Tinta et al., 2020). This indicates that MAOM offers a more immediately accessible pool of labile nutrients for microbial uptake.

Despite these differences, both OM sources share a remarkably similar amino acid profile: glycine and taurine dominate the DFAA pools of both MAOM (53.6 ± 8.3% and 8.0 ± 1.0%) and detritus-derived DOM (41.9% and 37.8%, respectively; Tinta et al., 2020). This consistency highlights their central roles in jellyfish physiology and underscores their importance as microbial substrates during jellyfish bloom events.

However, the high lability of MAOM comes at a cost. Its elevated C:N ratio (6.7 ± 1.0) compared to detritus DOM (3.4 ± 0.1) and bacterial biomass (5.2) likely imposes nitrogen limitation on microbial consumers. This imbalance is reflected in the lower microbial growth efficiency observed for MAOM (∼30%; Condon, Steinberg & Bronk, 2010) relative to detritus (∼65 ± 27%; Tinta et al., 2020)). As a result, MAOM primarily supports microbial respiration rather than biomass production, enhancing C remineralisation and stimulating the release of inorganic nutrients—such as DIN and PO_4_^3^−^^—which may indirectly support primary producers (Fig. 4). In contrast, detritus OM supports greater microbial growth, offering a more nutrient-balanced substrate that promotes biomass production over respiration.

Together, these findings demonstrate that MAOM and jellyfish detritus—though both products of jellyfish blooms—play complementary roles in the marine C and nutrient cycles. While MAOM rapidly stimulates microbial activity and nutrient regeneration, detritus provides a more sustained and productive microbial response, contributing to longer-term C processing. These distinct but interconnected pathways underscore the broader ecological influence of jellyfish and reinforce their role as active participants in coastal ecosystem functioning.

## Conclusions

Our study demonstrates that MAOM from *Aurelia* spp. represents a highly labile source of DOM that is rapidly metabolized by marine microbes. Glycine and taurine dominated the DFAA pool, reflecting their physiological roles as osmolytes in jellyfish. MAOM stoichiometric imbalance and rapid turnover primarily fuel microbial respiration rather than biomass production, with implications for oxygen dynamics in coastal ecosystems. Metagenomic evidence further revealed that MAOM degradation is facilitated by opportunistic copiotrophs with broad metabolic capacities, including specialized transporters for glycine and taurine. Comparisons with jellyfish detritus highlight the complementary roles of MAOM and detrital material in fuelling microbial activity: MAOM drives fast remineralisation and nutrient regeneration, whereas detritus supports more efficient microbial growth. Together, these findings illustrate that jellyfish blooms not only introduce large pulses of POM but also continuously release dissolved nutrients that shapes microbial community function and biogeochemical cycling in coastal seas. These findings underscore the importance of integrating jellyfish blooms into ecological models and ecosystem-based management strategies, offering critical data to support these efforts.

##  Supplemental Information

10.7717/peerj.20784/supp-1 Supplemental Information 1 Supplementary material

10.7717/peerj.20784/supp-2 Supplemental Information 2 Raw data from nutrient measurements Raw data of organic and inorganic nutrients (NH_4_^+^, NO_2_^−^, NO_3_^−^), PO_4_^3^−^^, TDN, DON, DOC, PON, POC, DFAAs) for the dialsis, leaching and degradation experiments. All concentrations are expressed in μ mol L^−1^ except form the DFAAs that are expressed in nmol L^−1^.

10.7717/peerj.20784/supp-3 Supplemental Information 3 Raw data from microbial measurements Raw data of microbial parameters, including prokaryotic heterotrophic production (PHP; µg C L^1^ h^1^), microbial abundance (MA; 10 y cells mL^1^), dissolved organic carbon (DOC; µg C L^1^), and total dissolved nitrogen (TDN; µg C LL^1^).

10.7717/peerj.20784/supp-4 Supplemental Information 4 Changes in particulate and dissolved nutrients during the leaching experiment Concentrations of DOC (A), TDN (B), POC (C) and PON (D) during the leaching experiments using frozen- and dry-MAOM.

10.7717/peerj.20784/supp-5Supplemental Information 5Changes in NO${}_{3}^{-}$ and NO${}_{2}^{-}$ during the degradation experimentNO${}_{3}^{-}$ (A) and NO${}_{2}^{-}$ (B) concentrations during the degradation experiment in frozen-, dry-MAOM, and control treatments. Lines represent means, with shaded areas indicating standard deviation.

10.7717/peerj.20784/supp-6 Supplemental Information 6 NMDS ordination of amino acid composition during the degradation experiment Results of the Nonmetric multidimensional scaling (NMDS) ordination of amino acid composition of the treatments (dry-MA OM, frozen- MAOM and control represented by circles, triangles and crosses, respectively) over time (0, 5, 10, 20, 30, 42 h; represented by the colour gradient from dark to light). The scores for dimensions 1 and 2 are shown in panel A while the contribution of amino acids for dimensions 1 and 2 are shown in panel B, with the length of the arrows indicating the importance of the respective amino acid to the dimension. GLU, glutamic acid; ASP, aspartic acid; SER, serine; HIS, histidine; GLY, glycine; THR, threonine; ARG, arginine; ALA, alanine; TAU, taurine; VAL, valine; ET, methionine; PHE, phenylalanine; ILE, isoleucine; LEU, leucine; LYS, lysine. Glutamine, asparagine and tyrosine are not shown as their contribution to the dimensions were negligible.

10.7717/peerj.20784/supp-7 Supplemental Information 7 Heatmap of COG functional categories at the end of the degradation experiment Heatmap of COG functional categories for the three analysed metagenomes (frozen-, dry- MAOM and control). For each row the values are scaled to make the differences more evident.

10.7717/peerj.20784/supp-8Supplemental Information 8Nutrient composition of the experimental mediaConcentration of dissolved and particulate nutrients (NH${}_{4}^{+}$, NO${}_{3}^{-}$, NO${}_{2}^{-}$, PO${}_{4}^{+}$, TDN, DOC, POC, PON and DFAA) in the ASW and the ASW:FSW (ratio 9:1).

10.7717/peerj.20784/supp-9 Supplemental Information 9 Comparison of nutrient leaching from dry- and frozen-MAOM Amount of particulate and dissolved organic nutrients (POC, PON, DOC, DON) that leached from dry- MAOM and frozen- MAOM within 1 h expressed in µmol per g of DW and *p* values from the Wilcoxon rank-sum tests evaluating the difference between the amount of nutrient released in each leaching experiment.

10.7717/peerj.20784/supp-10 Supplemental Information 10 Comparaison of DFAA composition from dry- and frozen-MAOM Percentages, concentrations and mean of the amino acids (GLU, glutamic acid; ASP, aspartic acid; ASN, asparagine; SER, serine; GLN, glutamine; HIS, histidine; GLY, glycine; THR, threonine; ARG, arginine; ALA, alanine; TAU, taurine; GABA, gammaaminobutyric acid; TYR, tyrosine; VAL, valine; MET, methionine; PHE, phenylalanine; ILE, isoleucine; LEU, leucine; LYS, lysine) released during the leaching experiment by dry- MAOM and frozen- MAOM .

10.7717/peerj.20784/supp-11Supplemental Information 11Nutrient release from the dialysis experimentCumulative amount of dissolved nutrients (NH${}_{4}^{+}$, NO${}_{3}^{-}$, NO${}_{2}^{-}$, PO${}_{4}^{+}$, DIN, TDN, DON, DOC, DFAA) that leached through a 1 kDa MWCO membrane tubing within 24 h expressed in µmol per g of dry weight (DW). The fraction <1 kDa represents low molecular weight compounds (LMW).

10.7717/peerj.20784/supp-12 Supplemental Information 12 Proportion of LMW DOM in dry-MAOM Cumulative amount of DOC, TDN and DON that leached through a 1 kDa MWCO membrane tubing within 24 h (LMW), that leached directly from dry- MAOM in 24 h and the percentage of LMW in dry- MAOM .

10.7717/peerj.20784/supp-13 Supplemental Information 13 DW content of dry-MOAM Wet weight (WW), dry weight (DW) and the percentage of DW in WW of 8 dry- MAOM samples.

10.7717/peerj.20784/supp-14Supplemental Information 14Statistical comparison of nutrient and microbial parameters between dry- and frozen-MAOM treatments*p* -values from Wilcoxon rank-sum tests comparing concentrations of dissolved inorganic nutrients (NH${}_{4}^{+}$, NO${}_{3}^{-}$, NO${}_{2}^{-}$, PO${}_{4}^{{3}^{-}}$) and microbial parameters (microbial abundance, PHP, DOC, and TDN) between dry-MAOM and frozen-MAOM treatments at each sampling time. “NA” indicates comparisons where all values were identical.

10.7717/peerj.20784/supp-15Supplemental Information 15Comparison of dissolved nutrient release from dry-MAOM and jellyfish detritusCumulative release of dissolved nutrients (NH${}_{4}^{+}$, NO${}_{3}^{-}$, NO${}_{2}^{-}$, PO${}_{4}^{{3}^{-}}$, DIN, TDN, DON, DOC, DFAA) from dry-MAOM (this study) and jellyfish detritus (Tinta et al., 2020) within 24 h, expressed in µmol g^1^ DW. Ratios represent mean values from detritus divided by mean values from dry-MAOM.

## Additional Information and Declarations

### DNA Deposition

The following information was supplied regarding the deposition of DNA sequences: The raw reads are available at the European Nucleotide Archive: PRJEB66855.

### Data Availability

The following information was supplied regarding data availability: The raw reads are available at the European Nucleotide Archive: PRJEB66855. The raw nutrient data, raw microbial data and the R code are available at GitHub and Zenodo: – https://github.com/Nathhub/Mucus_degradation_experiment/tree/v1.0.0. – Nathan Hubot. (2025). Nathhub/Mucus_degradation_experiment: Mucus degradation experiment v1.0 (v1.0.0). Zenodo. https://doi.org/10.5281/zenodo.17832253.

## References

[ref-1] Anderson MJ (2017). Permutational multivariate analysis of variance (PERMANOVA). Wiley StatsRef: statistics reference online.

[ref-2] Anderson MJ, Ellingsen KE, McArdle BH (2006). Multivariate dispersion as a measure of beta diversity. Ecology Letters.

[ref-3] Andrews S (2010). FastQC: a quality control tool for high throughput sequence data. http://www.bioinformatics.babraham.ac.uk/projects/fastqc.

[ref-4] Angel R (2012). Total nucleic acid extraction from soil. Protocol exchange, ahead of print, October 23.

[ref-5] Arnosti C (2011). Microbial extracellular enzymes and the marine carbon cycle. Annual Review of Marine Science.

[ref-6] Bakshani CR, Morales-Garcia AL, Althaus M, Wilcox MD, Pearson JP, Bythell JC, Burgess JG (2018). Evolutionary conservation of the antimicrobial function of mucus: a first defence against infection. NPJ Biofilms and Microbiomes.

[ref-7] Bayer SR, Countway PD, Wahle RA (2019). Developing an eDNA toolkit to quantify broadcast spawning events of the sea scallop placopecten magellanicus: moving beyond fertilization assays. Marine Ecology Progress Series.

[ref-8] Benner R (2002). Chemical composition and reactivity. Biogeochemistry of marine dissolved organic matter.

[ref-9] Berggren M, Laudon H, Haei M, Ström L, Jansson M (2010). Efficient aquatic bacterial metabolism of dissolved low-molecular-weight compounds from terrestrial sources. The ISME Journal.

[ref-10] Boysen AK, Durham BP, Kumler W, Key RS, Heal KR, Carlson LT, Groussman RD, Armbrust EV, Ingalls AE (2022). Glycine betaine uptake and metabolism in marine microbial communities. Environmental Microbiology.

[ref-11] Brotz L, Angel DL, Enrique-Navarro IDA, Lauritano C, Thibault D, Prieto L (2024). Rhizostomes as a resource: the expanding exploitation of jellyfish by humans. Advances in Marine Biology.

[ref-12] Cantalapiedra CP, Hernández-Plaza A, Letunic I, Bork P, Huerta-Cepas J (2021). eggNOG-Mapper v2: functional annotation, orthology assignments, and domain prediction at the metagenomic scale. Molecular Biology and Evolution.

[ref-13] Celussi M, Manna V, Banchi E, Fonti V, Bazzaro M, Flander-Putrle V, Klun K, Kralj M, Orel N, Tinta T (2024). Annual recurrence of prokaryotic climax communities in shallow waters of the North Mediterranean. Environmental Microbiology.

[ref-14] Clifford EL, Varela MM, De Corte D, Bode A, Ortiz V, Herndl GJ, Sintes E (2019). Taurine is a major carbon and energy source for marine prokaryotes in the North Atlantic Ocean off the Iberian Peninsula. Microbial Ecology.

[ref-15] Cole JR, Wang Q, Fish JA, Chai B, McGarrell DM, Sun Y, Brown CT, Porras-Alfaro A, Kuske CR, Tiedje JM (2014). Ribosomal database project: data and tools for high throughput rRNA analysis. Nucleic Acids Research.

[ref-16] Condon RH, Steinberg DK, Bronk DA (2010). Production of dissolved organic matter and inorganic nutrients by Gelatinous Zooplankton in the York River Estuary, Chesapeake Bay. Journal of Plankton Research.

[ref-17] Condon RH, Steinberg DK, Del Giorgio PA, Bouvier TC, Bronk DA, Graham WM, Ducklow HW (2011). Jellyfish blooms result in a major microbial respiratory sink of carbon in marine systems. Proceedings of the National Academy of Sciences of the United States of America.

[ref-18] Cordero H, Cuesta A, Meseguer J, Esteban MÁ (2016). Changes in the levels of humoral immune activities after storage of Gilthead Seabream (*Sparus Aurata*) Skin Mucus. Fish & Shellfish Immunology.

[ref-19] Costas-Selas C, Martínez-García S, Logares R, Hernández-Ruiz M, Teira E (2023). Role of bacterial community composition as a driver of the small-sized phytoplankton community structure in a productive coastal system. Microbial Ecology.

[ref-20] Dinasquet J, Kragh T, Schrøter M-L, Søndergaard M, Riemann L (2013). Functional and compositional succession of bacterioplankton in response to a gradient in bioavailable dissolved organic carbon. Environmental Microbiology.

[ref-21] Edgar RC (2010). Search and clustering orders of magnitude faster than BLAST. Bioinformatics.

[ref-22] Eilers H, Pernthaler J, Amann R (2000). Succession of pelagic marine bacteria during enrichment: a close look at cultivation-induced shifts. Applied and Environmental Microbiology.

[ref-23] Eren AM, Kiefl E, Shaiber A, Veseli I, Miller SE, Schechter MS, Fink I, Pan JN, Yousef M, Fogarty EC, Trigodet F, Watson AR, Esen ÖC, Moore RM, Clayssen Q, Lee MD, Kivenson V, Graham ED, Merrill BD, Karkman A, Blankenberg D, Eppley JM, Sjödin A, Scott JJ, Vázquez-Campos X, McKay LJ, McDaniel EA, Stevens SLR, Anderson RE, Fuessel J, Fernandez-Guerra A, Maignien L, Delmont TO, Willis AD (2021). Community-led, integrated, reproducible multi-omics with Anvi’o. Nature Microbiology.

[ref-24] Fadeev E, Hennenfeind JH, Amano C, Zhao Z, Klun K, Herndl GJ, Tinta T (2024). Bacterial degradation of ctenophore mnemiopsis leidyi organic matter. mSystems.

[ref-25] Feller WF, Henslee JG, Kinders RJ, Manderino GL, Tomita JT, Rittenhouse HG (1990). Mucin glycoproteins as tumor markers. Immunology Series.

[ref-26] Giering SLC, Evans C (2022). Overestimation of prokaryotic production by leucine incorporation—and how to avoid it. Limnology and Oceanography.

[ref-27] Girard L, Lood C, De Mot R, Van Noort V, Baudart J (2023). Genomic diversity and metabolic potential of marine pseudomonadaceae. Frontiers in Microbiology.

[ref-28] Glover JS, Ticer TD, Engevik MA (2022). Characterizing the mucin-degrading capacity of the human gut microbiota. Scientific Reports.

[ref-29] Godwin CM, Cotner JB (2015). Aquatic heterotrophic bacteria have highly flexible phosphorus content and biomass stoichiometry. The ISME Journal.

[ref-30] Graham WM, Gelcich S, Robinson KL, Duarte CM, Brotz L, Purcell JE, Madin LP, Mianzan H, Sutherland KR, Uye S, Pitt KA, Lucas CH, Bøgeberg M, Brodeur RD, Condon RH (2014). Linking human well-being and jellyfish: ecosystem services, impacts, and societal responses. Frontiers in Ecology and the Environment.

[ref-31] Guy-Haim T, Rubin-Blum M, Rahav E, Belkin N, Silverman J, Sisma-Ventura G (2020). The effects of decomposing invasive jellyfish on biogeochemical fluxes and microbial dynamics in an ultra-oligotrophic sea. Biogeosciences.

[ref-32] Haber M, Roth Rosenberg D, Lalzar M, Burgsdorf I, Saurav K, Lionheart R, Lehahn Y, Aharonovich D, Gómez-Consarnau L, Sher D, Krom MD, Steindler L (2022). Spatiotemporal variation of microbial communities in the ultra-oligotrophic Eastern Mediterranean Sea. Frontiers in Microbiology.

[ref-33] Hansell DA (1993). Results and observations from the measurement of doc and don in seawater using a high-temperature catalytic oxidation technique. Marine Chemistry, Measurement of Dissolved Organic Carbon and Nitrogen in Natural Waters.

[ref-34] Hansson LJ, Norrman B (1995). Release of Dissolved Organic Carbon (DOC) by the Scyphozoan jellyfish Aurelia Aurita and its potential influence on the production of planktic bacteria. Marine Biology.

[ref-35] Hays GC, Doyle TK, Houghton JDR (2018). A paradigm shift in the trophic importance of jellyfish?. Trends in Ecology & Evolution.

[ref-36] Hubot N, Giering SLC, Lucas CH (2022). Similarities between the biochemical composition of jellyfish body and mucus. Journal of Plankton Research.

[ref-37] Hydes DJ, Aoyama M, Aminot A, Bakker K, Becker S, Coverly S, Daniel A, Dickson AG, Grosso O, Kerouel R, Van Ooijen J, Sato K, Tanhua T, Woodward EMS, Zhang JZ (2010). Determination of dissolved nutrients (N, P, SI) in seawater with high precision and inter-comparability using gas-segmented continuous flow analysers. The GO-SHIP Repeat Hydrography Manual: A Collection of Expert Reports and Guidelines, Version 1 (eds E.M. Hood, C.L. Sabine & B.M. Sloyan). UNESCO–IOC, Paris, France. IOCCP Report No. 14, ICPO Publication Series No. 134.

[ref-38] Jones BN, Pääbo S, Stein S (1981). Amino acid analysis and enzymatic sequence determination of peptides by an improved o-phthaldialdehyde precolumn labeling procedure. Journal of Liquid Chromatography.

[ref-39] Kikuchi G, Motokawa Y, Yoshida T, Hiraga K (2008). Glycine cleavage system: reaction mechanism, physiological significance, and hyperglycinemia. Proceedings of the Japan Academy. Series B, Physical and Biological Sciences.

[ref-40] Kirchman D, K’nees E, Hodson R (1985). Leucine incorporation and its potential as a measure of protein synthesis by bacteria in natural aquatic systems. Applied and Environmental Microbiology.

[ref-41] Kogovšek T, Tinta T, Klun K, Malej A (2014). Jellyfish biochemical composition: importance of standardised sample processing. Marine Ecology Progress Series.

[ref-42] Kogovšek T, Vodopivec M, Raicich F, Uye S, Malej A (2018). Comparative analysis of the ecosystems in the Northern Adriatic Sea and the Inland Sea of Japan: can anthropogenic pressures disclose jellyfish outbreaks?. Science of The Total Environment.

[ref-43] Krueger F (2020). https://github.com/FelixKrueger/TrimGalore.

[ref-44] Langmead B, Salzberg SL (2012). Fast gapped-read alignment with bowtie 2. Nature Methods.

[ref-45] Legendre P, Legendre LFJ (1998). Numerical ecology.

[ref-46] Li H, Handsaker B, Wysoker A, Fennell T, Ruan J, Homer N, Marth G, Abecasis G, Durbin R, 1000 Genome Project Data Processing Subgroup (2009). The sequence alignment/map format and SAMtools. Bioinformatics.

[ref-47] Li D, Luo R, Liu C-M, Leung C-M, Ting H-F, Sadakane K, Yamashita H, Lam T-W (2016). MEGAHIT v1.0: a fast and scalable metagenome assembler driven by advanced methodologies and community practices. Methods, Pan-omics Analysis of Biological Data.

[ref-48] Lilley MKS, Beggs SE, Doyle TK, Hobson VJ, Stromberg KHP, Hays GC (2011). Global patterns of epipelagic gelatinous zooplankton biomass. Marine Biology.

[ref-49] Logares R, Sunagawa S, Salazar G, Cornejo-Castillo FM, Ferrera I, Sarmento H, Hingamp P, Ogata H, De Vargas C, Lima-Mendez G, Raes J, Poulain J, Jaillon O, Wincker P, Kandels-Lewis S, Karsenti E, Bork P, Acinas SG (2014). Metagenomic 16S rDNA Illumina tags are a powerful alternative to amplicon sequencing to explore diversity and structure of microbial communities. Environmental Microbiology.

[ref-50] Lucas CH, Loveridge A, Hubot ND (2024). Jellyfish in coastal ecosystems: advances in our understanding of population drivers, role in biogeochemical cycling, and socio-economic impacts. Treatise on estuarine and coastal science.

[ref-51] Luo JY, Condon RH, Stock CA, Duarte CM, Lucas CH, Pitt KA, Cowen RK (2020). Gelatinous zooplankton-mediated carbon flows in the global oceans: a data-driven modeling study. Global Biogeochemical Cycles.

[ref-52] Lynam C, Gibbons MJ, Axelsen BE, Sparks CAJ, Coetzee J, Heywood BG, Brierley AS (2006). Jellyfish overtake fish in a heavily fished ecosystem. Current Biology: CB.

[ref-53] Marini M, Grilli F (2023). The role of nitrogen and phosphorus in eutrophication of the Northern Adriatic Sea: history and future scenarios. Applied Sciences.

[ref-54] Merivaara A, Zini J, Koivunotko E, Valkonen S, Korhonen O, Fernandes FM, Yliperttula M (2021). Preservation of biomaterials and cells by freeze-drying: change of paradigm. Journal of Controlled Release.

[ref-55] Merquiol L, Romano G, Ianora A, D’Ambra I (2019). Biotechnological applications of scyphomedusae. Marine Drugs.

[ref-56] Mikheenko A, Prjibelski A, Saveliev V, Antipov D, Gurevich A (2018). Versatile genome assembly evaluation with QUAST-LG. Bioinformatics.

[ref-57] Oksanen J, Kindt R, Legendre P, Minchin PR, O’hara RB, Simpson GL, Solymos P, Stevens MHH, Wagner H (2013).

[ref-58] Orel N, Fadeev E, Klun K, Ličer M, Tinta T, Turk V (2021). Bacterial indicators are ubiquitous members of pelagic microbiome in anthropogenically impacted coastal ecosystem. Frontiers in Microbiology.

[ref-59] Pitt KA, Duarte CM, Lucas CH, Sutherland KR, Condon RH, Mianzan H, Purcell JE, Robinson KL, Uye S-I (2013). Jellyfish body plans provide allometric advantages beyond low carbon content. PLOS ONE.

[ref-60] Pitt KA, Welsh DT, Condon RH (2009). Influence of jellyfish blooms on carbon, nitrogen and phosphorus cycling and plankton production. Hydrobiologia.

[ref-61] Podbielski I, Hiebenthal C, Hajati M-C, Bock C, Bleich M, Melzner F (2022). Capacity for cellular osmoregulation defines critical salinity of marine invertebrates at low salinity. Frontiers in Marine Science.

[ref-62] Porter KG, Feig YS (1980). The use of DAPI for identifying and counting aquatic microflora1. Limnology and Oceanography.

[ref-63] R Core Team (2022). https://www.R-project.org/.

[ref-64] Redfield AC, Ketchum BH, Richards FA, Hill MN (1963). The influence of organisms on the composition of seawater. The Sea.

[ref-65] Richardson AJ, Bakun A, Hays GC, Gibbons MJ (2009). The jellyfish joyride: causes, consequences and management responses to a more gelatinous future. Trends in Ecology & Evolution.

[ref-66] Ríos AF, Fraga F, Pérez FF, Figueiras FG (1998). Chemical composition of phytoplankton and particulate organic matter in the Ría de Vigo (NW Spain). Scientia Marina.

[ref-67] Savoca S, DiFresco D, Alesci A, Capillo G, Spanò N (2022). Mucus secretions in Cnidarian, an ecological, adaptive and evolutive tool. Advances in Oceanography and Limnology.

[ref-68] Smith DC, Azam F (1992). A simple, economical method for measuring bacterial protein synthesis rates in seawater using 3H-leucine. https://www.semanticscholar.org/paper/A-simple%2C-economical-method-for-measuring-bacterial-Smith-Azam/de4d1a5b9a27b6ed7f982976c60c85175a4e08b7.

[ref-69] Tatusov RL, Natale DA, Garkavtsev IV, Tatusova TA, Shankavaram UT, Rao BS, Kiryutin B, Galperin MY, Fedorova ND, Koonin EV (2001). The COG database: new developments in phylogenetic classification of proteins from complete genomes. Nucleic Acids Research.

[ref-70] Tinta T, Klun K, Herndl GJ (2021). The importance of jellyfish–microbe interactions for biogeochemical cycles in the ocean. Limnology and Oceanography.

[ref-71] Tinta T, Vojvoda J, Mozetič P, Talaber I, Vodopivec M, Malfatti F, Turk V (2015). Bacterial community shift is induced by dynamic environmental parameters in a changing coastal ecosystem (Northern Adriatic, Northeastern Mediterranean Sea)–a 2-year time-series study. Environmental Microbiology.

[ref-72] Tinta T, Zhao Z, Bayer B, Herndl GJ (2023). Jellyfish detritus supports niche partitioning and metabolic interactions among pelagic marine bacteria. Microbiome.

[ref-73] Tinta T, Zhao Z, Escobar A, Klun K, Bayer B, Amano C, Bamonti L, Herndl GJ (2020). Microbial processing of jellyfish detritus in the ocean. Frontiers in Microbiology.

[ref-74] Vadstein O, Olsen LM, Busch A, Andersen T, Reinertsen HR (2003). Is phosphorus limitation of planktonic heterotrophic bacteria and accumulation of degradable DOC a normal phenomenon in phosphorus-limited systems? A microcosm study. FEMS Microbiology Ecology.

[ref-75] Ventura M (2006). Linking biochemical and elemental composition in freshwater and marine crustacean zooplankton. Marine Ecology Progress Series.

[ref-76] Vilibić I, Dunić N, Peharda M (2022). Near-surface ocean temperature variations across temporal scales in the Coastal Eastern Adriatic. Continental Shelf Research.

[ref-77] Welborn J, Manahan D (1995). Taurine metabolism in larvae of marine invertebrate molluscs (Bilvalvia, Gastropoda). Journal of Experimental Biology.

[ref-78] Wright RM, Le Quéré C, Buitenhuis E, Pitois S, Gibbons MJ (2021). Role of jellyfish in the plankton ecosystem revealed using a global ocean biogeochemical model. Biogeosciences.

