# Supplementary material for: Jellyfish mucus-derived organic matter as a source of labile nutrients for the ambient microbial community
Source: PeerJ. 2026 Feb 12;14:e20784. doi: 10.7717/peerj.20784 (PMC12906709; doi:10.7717/peerj.20784)
Supplement: Supplemental Information 1 [file peerj-14-20784-s001.docx]

**Supplementary Methods**

Jellyfish Mucus-Derived Organic Matter as a Source of Labile Nutrients for the Ambient Microbial Community

*Dialysis*

Dialysis of dry-MAOM was performed using Spectra/Por 7 Membrane tubing (Sulphur and Heavy Metal Free Spectrum) with a molecular weight cut-off (MWCO) of 1 kDa using a duplicate sample to determine the ratio between the HMW and the LMW. Dialysis membranes were prepared and used according to the manufacturer’s instructions (Spectrum) and sealed with Spectrum™ Universal Dialysis Tubing Closures (Spectrum). For each duplicate, 1 g of MAOM DW was re-hydrated in 25 mL of Milli-Q water to mimic the average wet weight of the collected fresh jelly-OM and poured into a dialysis bag. The dialysis bags were submerged in an acid-cleaned and combusted glass beaker filled with 500 mL Milli-Q water and stirred at low speed on a magnetic stirrer. Dialysis of all duplicates was performed at 4 °C in the dark for 24 h by changing Milli-Q water after 2, 4, 9 and 24 h until salinity reached 0. Salinity was measured using a refractometer. The control consisted of an empty dialysis membrane submerged in Milli-Q water. At each time point the dialysate was sampled for determining the concentrations of DOC, TDN, DFAA, PO_4_^-^, NH_4_^+^, NO_3_^-^ and NO_2_^-^ . At the end of the dialysis, the dialysate of each duplicate was sampled and analysed for possible bacterial contamination. Control data for DOC and TDN were missing due to tube breakage during freezing. Therefore, the control corrections, accounting for the amount of DOC and TDN leached by the bag and clip, were performed using values from (Tinta et al., 2020) from the same experimental setup (DOC: 178.67 µM, TDN: 15.19 µM).

# References

Tinta, T., Zhao, Z., Escobar, A., Klun, K., Bayer, B., Amano, C., et al. (2020). Microbial Processing of Jellyfish Detritus in the Ocean. *Front. Microbiol.* 11. doi: 10.3389/fmicb.2020.590995
