# Supplementary figures and images for: Jellyfish mucus-derived organic matter as a source of labile nutrients for the ambient microbial community
Source: PeerJ. 2026 Feb 12;14:e20784. doi: 10.7717/peerj.20784 (PMC12906709; doi:10.7717/peerj.20784)

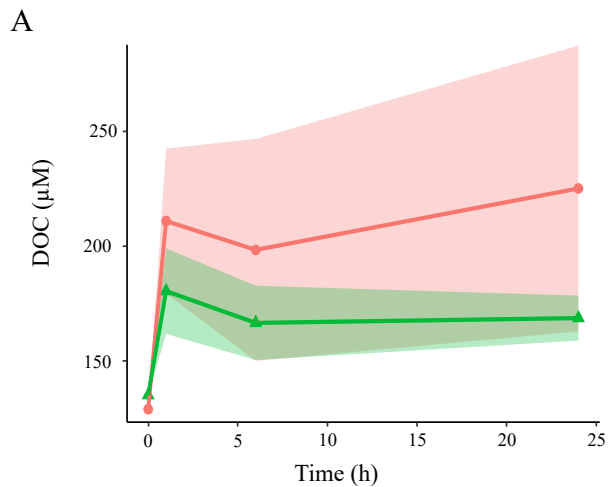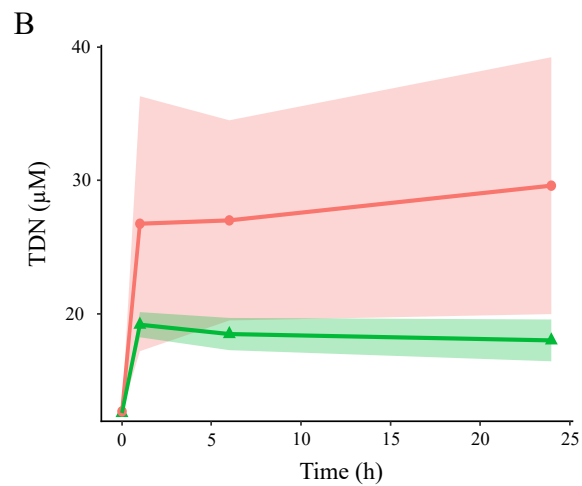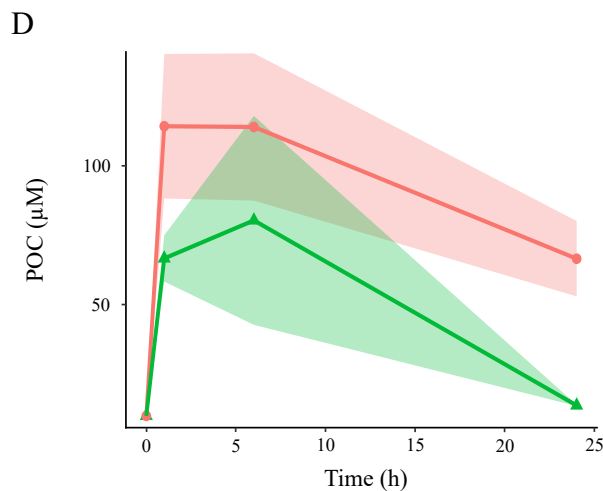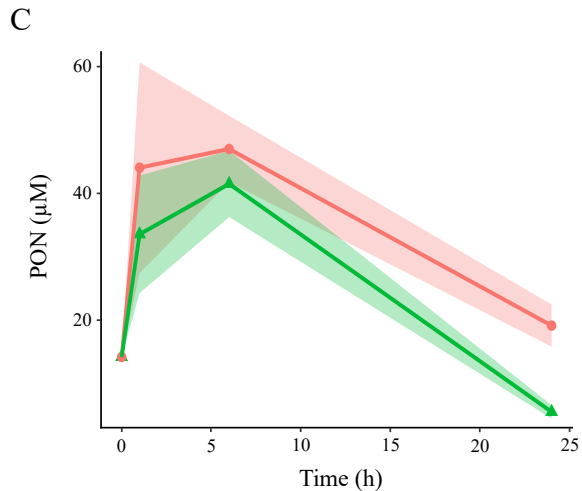

Treatment      ● Frozen-MAOM      ▲ Dry-MAOM

Supplement: Supplemental Information 4 — Concentrations of DOC (A), TDN (B), POC (C) and PON (D) during the leaching experiments using frozen- and dry-MAOM. [file peerj-14-20784-s004.pdf]

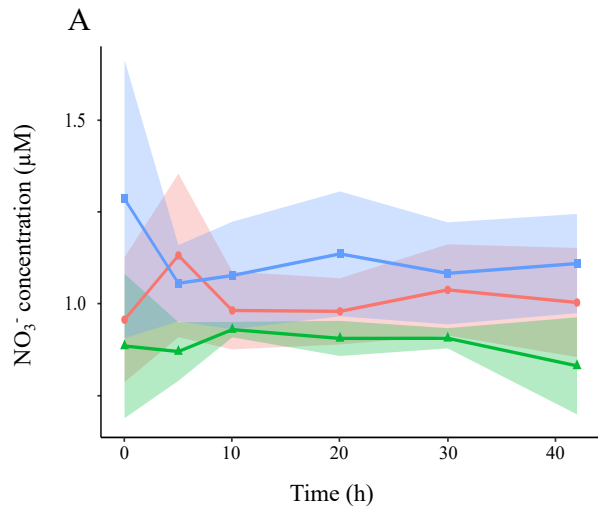

Treatment      ■ Frozen-MAOM      ■ Dry-MAOM      ■ Control

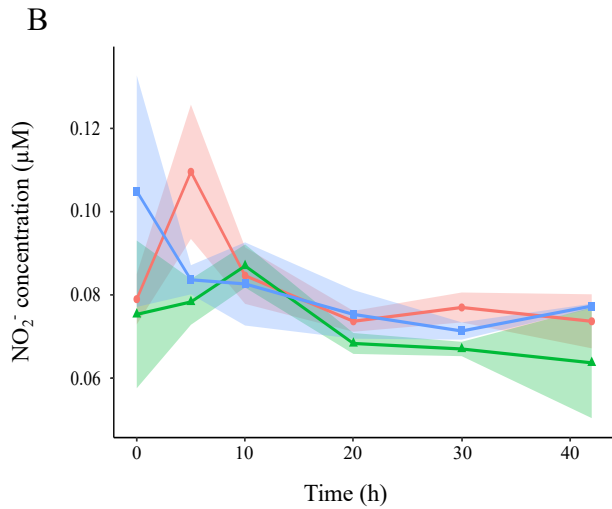

Supplement: Supplemental Information 5 — NO${}_{3}^{-}$3− (A) and NO${}_{2}^{-}$2− (B) concentrations during the degradation experiment in frozen-, dry-MAOM, and control treatments. Lines represent means, with shaded areas indicating standard deviation. [file peerj-14-20784-s005.pdf]

A

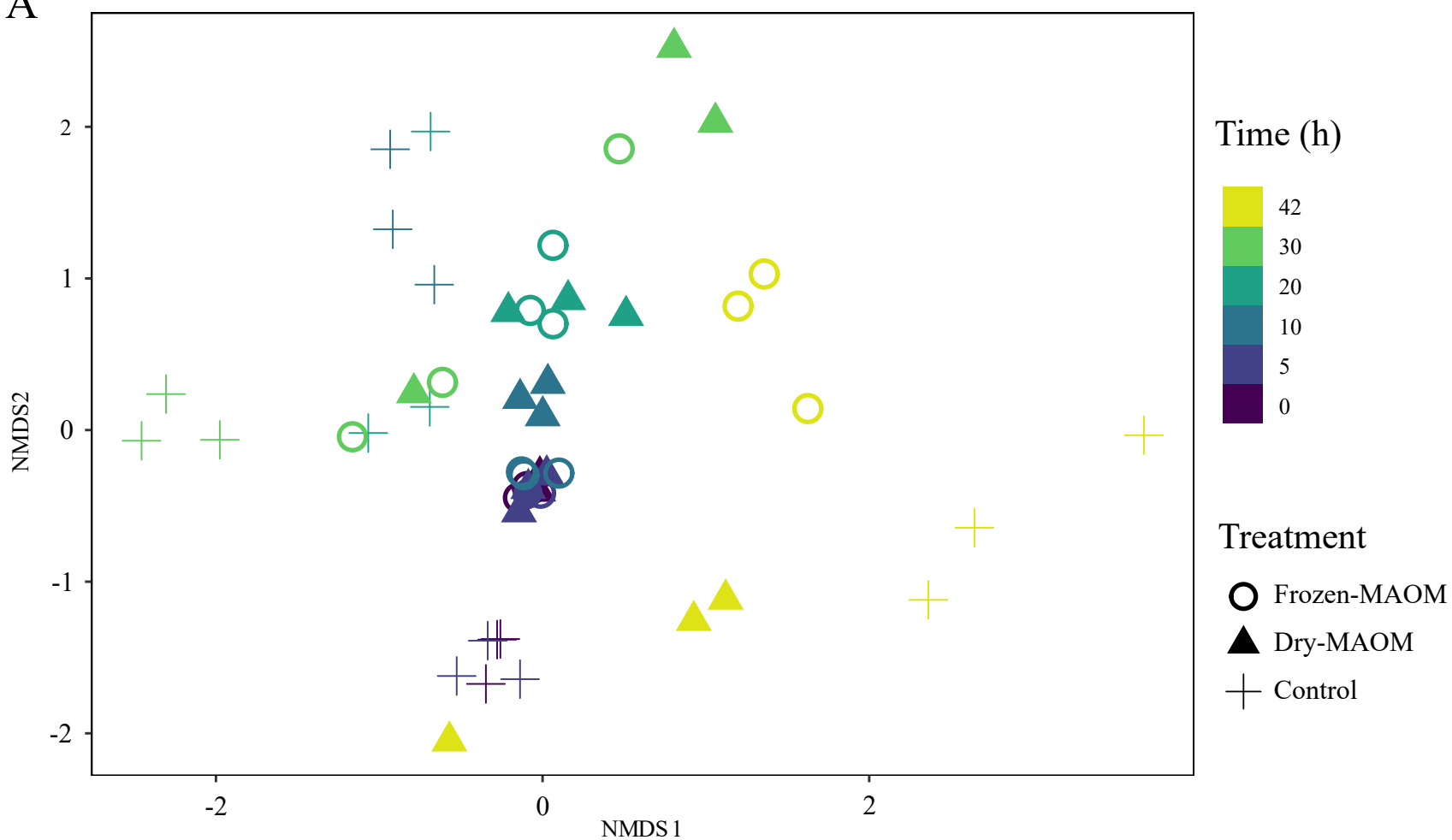

B

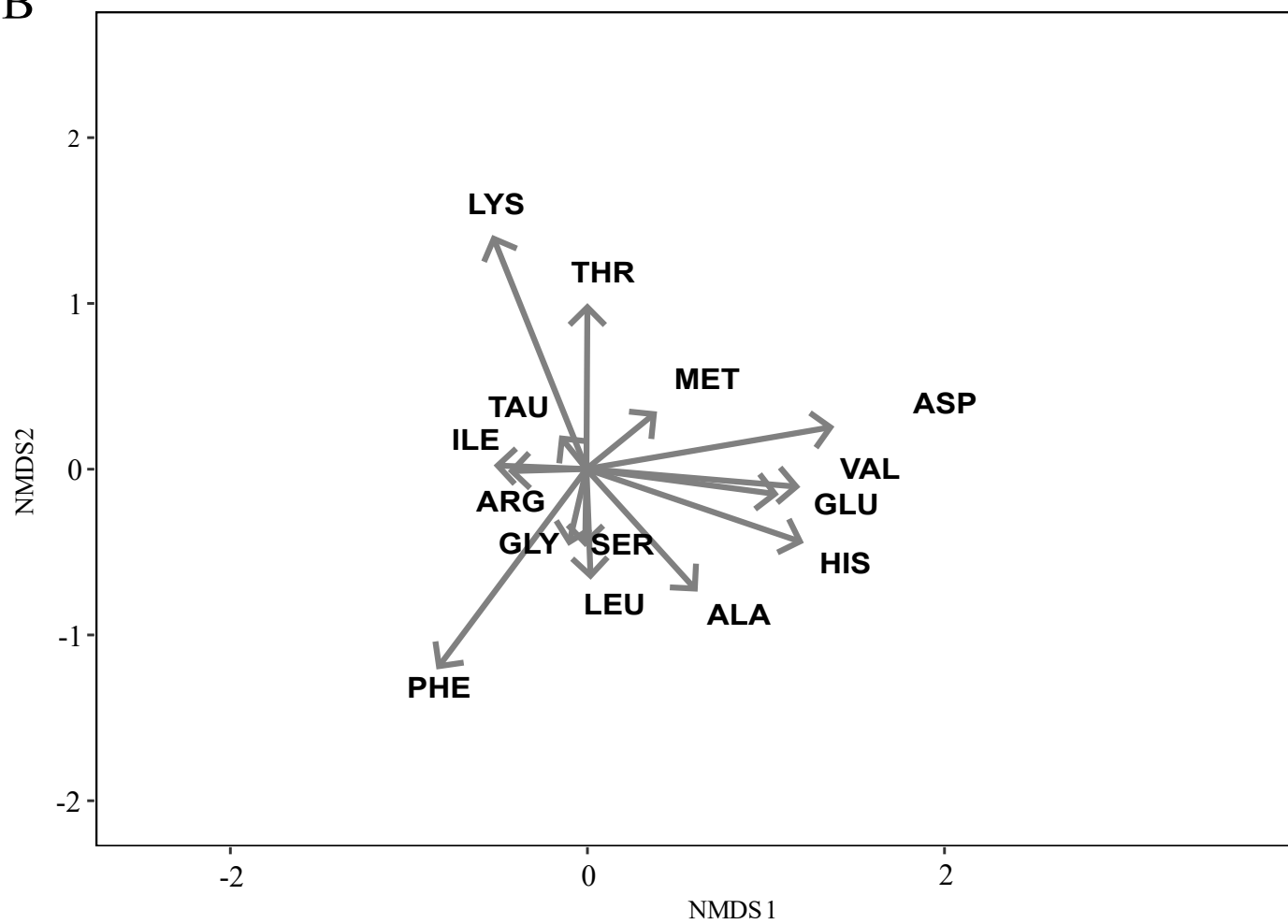

Supplement: Supplemental Information 6 — Results of the Nonmetric multidimensional scaling (NMDS) ordination of amino acid composition of the treatments (dry-MA OM, frozen- MAOM and control represented by circles, triangles and crosses, respectively) over time (0, 5, 10, 20, 30, 42 h; represented by the colour gradient from dark to light). The scores for dimensions 1 and 2 are shown in panel A while the contribution of amino acids for dimensions 1 and 2 are shown in panel B, with the length of the arrows indicating the importance of the respective amino acid to the dimension. GLU, glutamic acid; ASP, aspartic acid; SER, serine; HIS, histidine; GLY, glycine; THR, threonine; ARG, arginine; ALA, alanine; TAU, taurine; VAL, valine; ET, methionine; PHE, phenylalanine; ILE, isoleucine; LEU, leucine; LYS, lysine. Glutamine, asparagine and tyrosine are not shown as their contribution to the dimensions were negligible. [file peerj-14-20784-s006.pdf]

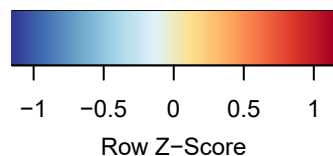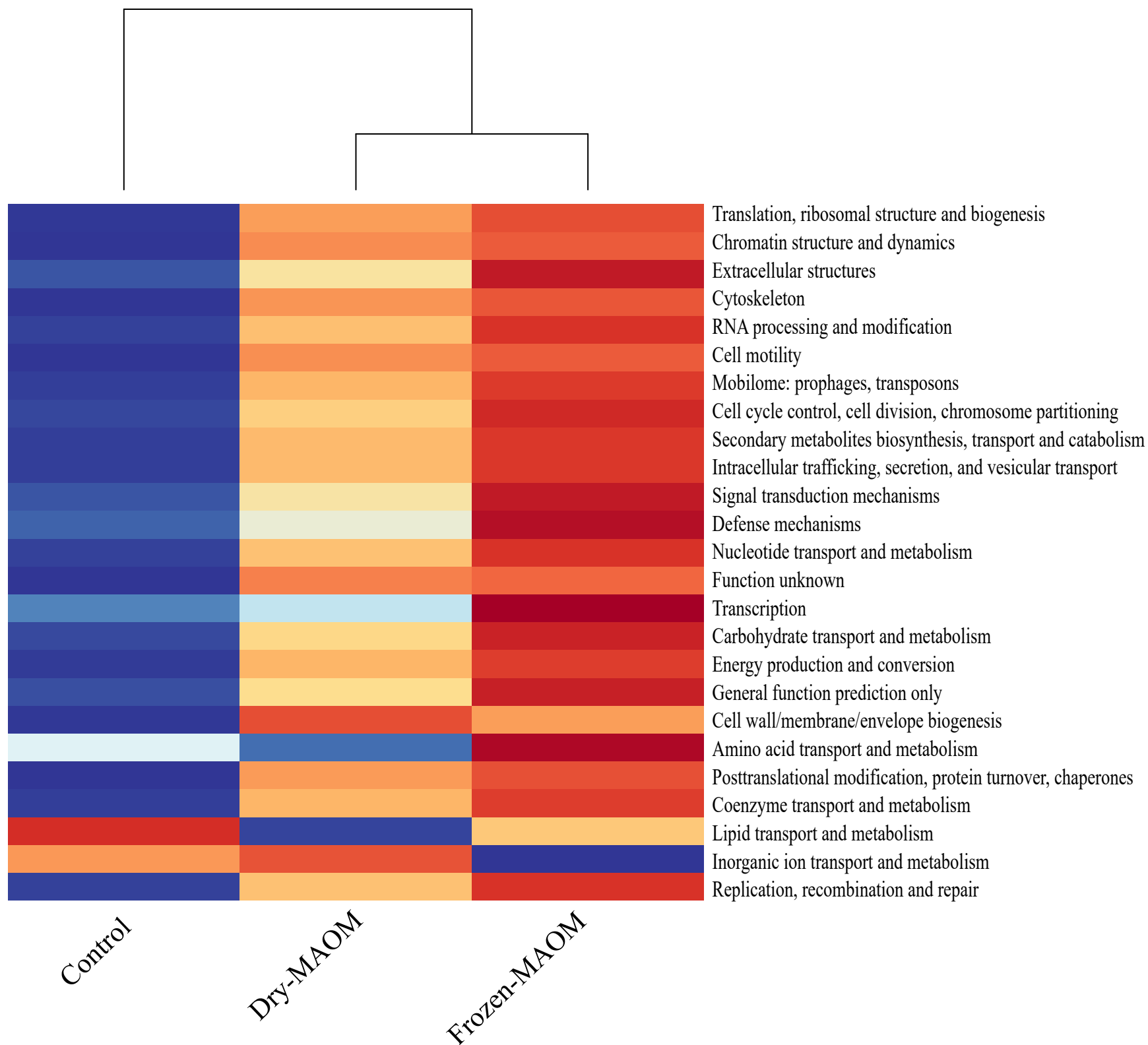

Supplement: Supplemental Information 7 — Heatmap of COG functional categories for the three analysed metagenomes (frozen-, dry- MAOM and control). For each row the values are scaled to make the differences more evident. [file peerj-14-20784-s007.pdf]
