# Supplementary material for: Jellyfish mucus-derived organic matter as a source of labile nutrients for the ambient microbial community
Source: PeerJ. 2026 Feb 12;14:e20784. doi: 10.7717/peerj.20784 (PMC12906709; doi:10.7717/peerj.20784)
Supplement: Supplemental Information 8 — Concentration of dissolved and particulate nutrients (NH${}_{4}^{+}$4+, NO${}_{3}^{-}$3−, NO${}_{2}^{-}$2−, PO${}_{4}^{+}$4+, TDN, DOC, POC, PON and DFAA) in the ASW and the ASW:FSW (ratio 9:1). [file peerj-14-20784-s008.docx]

|  | ASW | ASW:FSW |
| --- | --- | --- |
|  | (µmol L^-1^) | (µmol L^-1^) |
| NH_4_^+^ | 3.4 ± 0.2 | 3.0 ± 0.2 |
| NO_3_^-^ | 1.3 ± 0.0 | 1.0 ± 0.3 |
| NO_2_^-^ | 0.1 ± 0.0 | 0.1 ± 0.0 |
| PO_4_^3-^ | 0.1 ± 0.0 | 0.1 ± 0.0 |
| TDN | 12.6 ± 0.1 | 10.2 ± 1.1 |
| DOC | 132.0 ± 3.3 | 125.2 ± 15.1 |
| POC | 9.9 ± 0.0 | 11.3 ± 5.5 |
| PON | 14.2 ± 0.0 | 19.6 ± 10.2 |
| DFAA | 0.1 ± 0.0 | 0.1 ± 0.0 |
