# Supplementary material for: Jellyfish mucus-derived organic matter as a source of labile nutrients for the ambient microbial community
Source: PeerJ. 2026 Feb 12;14:e20784. doi: 10.7717/peerj.20784 (PMC12906709; doi:10.7717/peerj.20784)
Supplement: Supplemental Information 9 — Amount of particulate and dissolved organic nutrients (POC, PON, DOC, DON) that leached from dry- MAOM and frozen- MAOM within 1 h expressed in µmol per g of DW and p values from the Wilcoxon rank-sum tests evaluating the difference between the amount of nutrient released in each leaching experiment. [file peerj-14-20784-s009.docx]

|  | Dry-MAOM | Frozen-MAOM | Wilcoxon test |
| --- | --- | --- | --- |
|  | (µmol g^-1^ DW h^-1^) | (µmol g^-1^ DW h^-1^) | *p* |
| POC | 226.8 ± 33.4 | 317.2 ± 104.1 | 0.33 |
| PON | 77.4 ± 37.0 | 119.4 ± 66.5 | 0.67 |
| DOC | 134.0 ± 39.1 | 383.6 ± 248.3 | 0.33 |
| DON | 17.9 ± 3.9 | 62.8 ± 35.8 | 0.33 |
