# Supplementary material for: Jellyfish mucus-derived organic matter as a source of labile nutrients for the ambient microbial community
Source: PeerJ. 2026 Feb 12;14:e20784. doi: 10.7717/peerj.20784 (PMC12906709; doi:10.7717/peerj.20784)
Supplement: Supplemental Information 10 — Percentages, concentrations and mean of the amino acids (GLU, glutamic acid; ASP, aspartic acid; ASN, asparagine; SER, serine; GLN, glutamine; HIS, histidine; GLY, glycine; THR, threonine; ARG, arginine; ALA, alanine; TAU, taurine; GABA, gammaaminobutyric acid; TYR, tyrosine; VAL, valine; MET, methionine; PHE, phenylalanine; ILE, isoleucine; LEU, leucine; LYS, lysine) released during the leaching experiment by dry- MAOM and frozen- MAOM . [file peerj-14-20784-s010.docx]

|  | Dry-MAOM | | Frozen-MAOM | |  |
| --- | --- | --- | --- | --- | --- |
|  | Percent  (%) | Concentration  (nM) | Percent  (%) | Concentration  (nM) | Mean  (%) |
| GLU | 0.7 ± 0.2 | 109.5 ± 13.3 | 0.3 ± 0.2 | 148.7 ± 154.1 | 0.5 |
| ASP | 2.9 ± 0.5 | 433.7 ± 6.6 | 4.4 ± 1.7 | 1370.9 ± 447.9 | 3.6 |
| ASN | 0.2 ± 0.0 | 31.1 ± 0.5 | 0.2 ± 0.0 | 78.8 ± 62.6 | 0.2 |
| SER | 1.3 ± 0.1 | 199.5 ± 15.7 | 1.6 ± 0.1 | 588.5 ± 406.7 | 1.5 |
| GLN | 0.5 ± 0.0 | 78.2 ± 5.8 | 0.3 ± 0.1 | 110.7 ± 93.7 | 0.4 |
| HIS | 0.0 ± 0.0 | 0.0 ± 0.0 | 0.7 ± 1.0 | 133.6 ± 188.9 | 0.4 |
| GLY | 60.5 ± 4.2 | 9084.4 ± 862.1 | 46.8 ± 1.8 | 16578.2 ± 10550.9 | 53.6 |
| THR | 0.9 ± 1.2 | 157.5 ± 201.3 | 0.7 ± 0.7 | 316.0 ± 397.3 | 0.8 |
| ARG | 3.1 ± 0.2 | 471.8 ± 111.3 | 4.0 ± 0.6 | 1525.7 ± 1189.3 | 3.6 |
| ALA | 2.5 ± 1.0 | 363.1 ± 86.0 | 4.0 ± 0.6 | 1494.6 ± 1152.8 | 3.2 |
| TAU | 8.5 ± 0.9 | 1273.0 ± 75.7 | 7.5 ± 1.1 | 2563.4 ± 1403.6 | 8.0 |
| GABA | 5.0 ± 7.1 | 847.1 ± 1198.0 | 8.6 ± 1.3 | 2947.7 ± 1614.8 | 6.8 |
| TYR | 1.8 ± 0.1 | 268.4 ± 65.4 | 2.1 ± 0.2 | 737.4 ± 431.6 | 1.9 |
| VAL | 2.5 ± 0.4 | 378.2 ± 4.9 | 4.1 ± 1.5 | 1659.1 ± 1533.2 | 3.3 |
| MET | 1.4 ± 0.1 | 203.5 ± 19.0 | 2.3 ± 0.1 | 848.3 ± 582.2 | 1.8 |
| PHE | 0.4 ± 0.0 | 58.9 ± 6.7 | 0.9 ± 1.5 | 500.2 ± 748.2 | 0.6 |
| ILE | 1.8 ± 0.2 | 265.6 ± 8.7 | 2.7 ± 0.5 | 1040.7 ± 825.9 | 2.3 |
| LEU | 2.4 ± 0.4 | 351.1 ± 1.8 | 4.1 ± 0.8 | 1583.7 ± 1283.5 | 3.2 |
| LYS | 3.6 ± 0.5 | 532.5 ± 14.6 | 4.5 ± 0.4 | 1668.7 ± 1232.8 | 4.0 |
