# Supplementary material for: Jellyfish mucus-derived organic matter as a source of labile nutrients for the ambient microbial community
Source: PeerJ. 2026 Feb 12;14:e20784. doi: 10.7717/peerj.20784 (PMC12906709; doi:10.7717/peerj.20784)
Supplement: Supplemental Information 11 — Cumulative amount of dissolved nutrients (NH${}_{4}^{+}$4+, NO${}_{3}^{-}$3−, NO${}_{2}^{-}$2−, PO${}_{4}^{+}$4+, DIN, TDN, DON, DOC, DFAA) that leached through a 1 kDa MWCO membrane tubing within 24 h expressed in µmol per g of dry weight (DW). The fraction <1 kDa represents low molecular weight compounds (LMW). [file peerj-14-20784-s011.docx]

|  | LMW  (µmol gDW^-1^ d^-1^) |
| --- | --- |
| NH_4_^+^ | 2.0 ± 0.3 |
| NO_3_^-^ | 0.8 ± 0.3 |
| NO_2_^-^ | <LOD |
| PO_4_^3-^ | 1.5 ± 0.3 |
| DIN | 2.8 ± 0.6 |
| TDN | 21.6 ± 3.7 |
| DON | 18.8 ± 3.1 |
| DOC | 57.5 ± 38.5 |
| DFAA | 9.7 ± 0.6 |
