# Supplementary material for: Jellyfish mucus-derived organic matter as a source of labile nutrients for the ambient microbial community
Source: PeerJ. 2026 Feb 12;14:e20784. doi: 10.7717/peerj.20784 (PMC12906709; doi:10.7717/peerj.20784)
Supplement: Supplemental Information 12 — Cumulative amount of DOC, TDN and DON that leached through a 1 kDa MWCO membrane tubing within 24 h (LMW), that leached directly from dry- MAOM in 24 h and the percentage of LMW in dry- MAOM . [file peerj-14-20784-s012.docx]

|  | LMW  (µmol gDW^−1^) | Dry-MAOM  (µmol gDW^−1^) | LMW in dry-MAOM  (%) |
| --- | --- | --- | --- |
| DOC | 57.5 ± 28.5 | 134.0 ± 39.1 | 48.1 ± 35.4 |
| TDN | 21.6 ± 3.7 | 21.7 ± 6.3 | ≈100 |
| DON | 18.8 ± 3.1 | 17.8 ± 3.9 | ≈100 |
