# Supplementary material for: Jellyfish mucus-derived organic matter as a source of labile nutrients for the ambient microbial community
Source: PeerJ. 2026 Feb 12;14:e20784. doi: 10.7717/peerj.20784 (PMC12906709; doi:10.7717/peerj.20784)
Supplement: Supplemental Information 13 — Wet weight (WW), dry weight (DW) and the percentage of DW in WW of 8 dry- MAOM samples. [file peerj-14-20784-s013.docx]

| Sample | WW (g) | DW (g) | %DW |
| --- | --- | --- | --- |
| 1 | 24.70 | 1.03 | 4.16 |
| 2 | 18.12 | 0.85 | 4.68 |
| 3 | 23.73 | 0.95 | 4.01 |
| 4 | 29.62 | 1.22 | 4.12 |
| 5 | 30.21 | 1.17 | 3.87 |
| 6 | 19.91 | 0.73 | 3.67 |
