# Supplementary material for: Jellyfish mucus-derived organic matter as a source of labile nutrients for the ambient microbial community
Source: PeerJ. 2026 Feb 12;14:e20784. doi: 10.7717/peerj.20784 (PMC12906709; doi:10.7717/peerj.20784)
Supplement: Supplemental Information 14 — p -values from Wilcoxon rank-sum tests comparing concentrations of dissolved inorganic nutrients (NH${}_{4}^{+}$4+, NO${}_{3}^{-}$3−, NO${}_{2}^{-}$2−, PO${}_{4}^{{3}^{-}}$43−) and microbial parameters (microbial abundance, PHP, DOC, and TDN) between dry-MAOM and frozen-MAOM treatments at each sampling time. “NA” indicates comparisons where all values were identical. [file peerj-14-20784-s014.docx]

| Time (h) | 0 | 5 | 10 | 20 | 30 | 42 |
| --- | --- | --- | --- | --- | --- | --- |
| NH_4_^+^ | 1 | 0.7 | 0.4 | 1 | 1 | 0.4 |
| NO_3_^-^ | 0.7 | 0.2 | 0.7 | 0.2 | 0.2 | 0.4 |
| NO_2_^-^ | 0.82 | 0.1 | 0.64 | 0.12 | 0.08 | 0.51 |
| PO_4_^3-^ | 0.4 | 0.27 | 0.51 | 0.1 | *NA* | 1 |
| MA | *NA* | 0.4 | 0.2 | 1 | 0.1 | 1 |
| PHP | *NA* | 0.2 | 0.1 | 0.4 | 0.4 | 0.2 |
| DOC | *NA* | 1 | 0.2 | 0.7 | 1 | 0.2 |
| TDN | *NA* | 0.7 | 0.2 | 0.7 | 1 | 0.2 |
