# Supplementary material for: Jellyfish mucus-derived organic matter as a source of labile nutrients for the ambient microbial community
Source: PeerJ. 2026 Feb 12;14:e20784. doi: 10.7717/peerj.20784 (PMC12906709; doi:10.7717/peerj.20784)
Supplement: Supplemental Information 15 — Cumulative release of dissolved nutrients (NH${}_{4}^{+}$4+, NO${}_{3}^{-}$3−, NO${}_{2}^{-}$2−, PO${}_{4}^{{3}^{-}}$43−, DIN, TDN, DON, DOC, DFAA) from dry-MAOM (this study) and jellyfish detritus (Tinta et al., 2020) within 24 h, expressed in µmol g1 DW. Ratios represent mean values from detritus divided by mean values from dry-MAOM. [file peerj-14-20784-s015.docx]

|  | Dry-MAOM | Dry-Detritus | Detritus/MAOM |
| --- | --- | --- | --- |
|  | (µmol gDW^−1^ d^−1^) | (µmol gDW^−1^ d^−1^) |  |
| NH_4_^+^ | 1.3 ± 2.7 | 4.5 ± 2.9 | 3.5 |
| NO_3_^-^ | 2.5 ± 0.3 | 0.3 ± 0.2 | 0.1 |
| NO_2_^-^ | 0.1 ± 0.1 | 0.1 ± 0.1 | 1 |
| PO_4_^+^ | 1.7 ± 0.1 | 2.2 ± 1.1 | 1.3 |
| DIN | 3.9 ± 2.3 | 4.8 ± 2.7 | 1.2 |
| TDN | 21.7 ± 6.3 | 67.6 ± 38.5 | 3.1 |
| DON | 17.9 ± 3.9 | 62.8 ± 35.8 | 3.5 |
| DOC | 134.0 ± 39.1 | 383.6 ± 248.3 | 2.9 |
| FDAA | 15.1 ± 2.5 | 35.9 ± 23.9 | 2.4 |
